# Cell transfection of purified cytolethal distending toxin B subunits allows comparing their nuclease activity while plasmid degradation assay does not

**DOI:** 10.1371/journal.pone.0214313

**Published:** 2019-03-28

**Authors:** Benoît J. Pons, Elisabeth Bezine, Mélissa Hanique, Valérie Guillet, Lionel Mourey, Johana Chicher, Teresa Frisan, Julien Vignard, Gladys Mirey

**Affiliations:** 1 INRA, UMR1331, Toxalim, Research Centre in Food Toxicology, Toulouse, France; 2 Université Toulouse III–Paul Sabatier (UPS), Toulouse, France; 3 Institut National Polytechnique de Toulouse, Toulouse, France; 4 Centre National de la Recherche Scientifique (CNRS), Institut de Pharmacologie et de Biologie Structurale (IPBS), Toulouse, France; 5 Plateforme protéomique Strasbourg Esplanade, Institut de Biologie Moléculaire et Cellulaire (IBMC), FRC1589 Strasbourg, France; 6 Department of Cell and Molecular Biology, Karolinska Institute, Stockholm, Sweden; Universidad de Costa Rica, COSTA RICA

## Abstract

The Cytolethal Distending Toxin (CDT) is produced by many pathogenic bacteria. CDT is known to induce genomic DNA damage to host eukaryotic cells through its catalytic subunit, CdtB. CdtB is structurally homologous to DNase I and has a nuclease activity, dependent on several key residues. Yet some differences between various CdtB subunit activities, and discrepancies between biochemical and cellular data, have been observed. To better characterise the role of CdtB in the induction of DNA damage, we affinity-purified wild-type and mutants of CdtB, issued from *E*. *coli* and *H*. *ducreyi*, under native and denaturing conditions. We then compared their nuclease activity by a classic *in vitro* assay using plasmid DNA, and two different eukaryotic assays–the first assay where host cells were transfected with a plasmid encoding CdtB, the second assay where host cells were directly transfected with purified CdtB. We show here that *in vitro* nuclease activities are difficult to quantify, whereas CdtB activities in host cells can be easily interpreted and confirmed the loss of function of the catalytic mutant. Our results highlight the importance of performing multiple assays while studying the effects of bacterial genotoxins, and indicate that the classic *in vitro* assay should be complemented with cellular assays.

## Introduction

The cytolethal distending toxin (CDT), a virulence factor that induces cellular distension, has been discovered in *Shigella* spp. [1], pathogenic *Escherichia coli* strains [1,2], and later found produced by many pathogenic Gram-negative bacteria, such as *Haemophilus ducreyi* [3], *Aggregatibacter actinomycetemcomitans* [4], *Campylobacter* spp. [2] and others [5]. CDT is able to induce DNA damage in eukaryotic cells, which leads to cell cycle arrest [4,6] and eventually senescence or cell death by apoptosis [6,7], when the damage is beyond repair. CDT is an AB_2_ toxin composed of three subunits, CdtA, CdtB and CdtC [8–10], encoded by an operon, with CdtB mediating the CDT cellular effects [11,12]. One exception is the typhoid toxin, produced by *Salmonella enterica* serovar Typhi, where CdtB is associated with PltA and PltB proteins [13].

CDT is produced by many Gram-negative bacteria and CDT genes exhibit some sequence variability, with the *cdtB* gene being the most conserved [14]. CdtB serves as the catalytic subunit of the CDT holotoxin, with sequence alignment analysis revealing some homology with the endo-/exo-nucleases and phosphatases (EEP) superfamily, including the deoxyribonuclease I (DNase I) [11,12,14]. The crystal structure of *H*. *ducreyi* CDT confirmed the CdtB structural homology with DNase I [10]. Moreover, the structure of the CdtB subunit is highly conserved, with the residues implicated in activity or 3D structure being strictly conserved [15,16]. Many purified CdtBs have been shown to present a nuclease activity by plasmid digestion assay [17–20], a test classically performed to study the nuclease activity of DNAse I [21]. This nuclease activity seems to be abolished when any conserved residue, important for the DNAse I catalytic activity, binding of the bivalent Mg2+ cation or binding to the DNA substrate, is mutated [12]. However, some discrepancies between CdtB activities, but also between biochemical and cellular data, have been noticed. Using plasmid digestion assays, the H261 residue of EcolCdtB, corresponding to the H252 DNase I catalytic residue, was described to be essential [12], in contrast to the homologous H274 residue in AactCdtB [22]. On the other hand, mutations in one of these two homologous residues abolished the CdtB-related defects, i.e. DNA damage and cell distension, when tested on cellular systems. In the same way, simultaneous mutations of the putative DNA binding residues R117, R144 and N201 of AactCdtB and HducCdtB alter the CDT-related cell cycle defects [10,23]. However, while the HducCdtB DNA binding mutant looses the ability to degrade plasmid DNA *in vitro*, the AactCdtB mutant shows enhanced nuclease activity in a similar assay. It is therefore important to carefully reassess the nuclease activity of the different CdtB mutants, especially by *in vitro* approaches, in order to accurately characterise their specific role in CDT toxicity.

Here, we aimed to better characterise the CdtB capacity to induce DNA damage. We evaluated nuclease activities *ex vivo*, using artificial ways to deliver CdtB to the nucleus, thus bypassing CdtA/CdtC-mediated recognition of target cells, endocytosis, and retrograde trafficking of CdtB that may differ between CDTs [24,25]. We first assessed the ability of wild-type (WT) and mutant CdtB subunits to induce DNA damage when ectopically expressed in eukaryotic cells. This assay allows bypassing any possible alteration of the intracellular trafficking of the holotoxin containing the mutant CdtB subunit, which may prevent proper nuclear translocation [24,25]. The WT CdtB induced DNA damage whilst the mutant did not, as assessed by phosphorylation of the early DNA damage marker γH2AX [26], thus confirming the loss of function of the catalytic mutant. In addition, we affinity-purified WT and catalytic mutants of CdtB, issued from *E*. *coli* and *H*. *ducreyi*, under native or denaturing conditions. We compared their nuclease activity by a classic *in vitro* assay, using supercoiled plasmid DNA, an assay currently used in the literature. Intriguingly, we show an identical *in vitro* activity for WT and mutant CdtB, independently of their bacterial origin or purification conditions. Finally, host cell DNA damage induced by direct transfection of the previously purified CdtB was measured. In contrast with the *in vitro* plasmid digestion, this second cellular assay displayed a clear difference between the WT and mutant CdtB and was shown to be quantitative. Our results indicate that, in order to study CdtB nuclease activity, the classic *in vitro* assay should be complemented with *ex vivo* experimental procedures, such as the protein transfection assay that we propose here.

## Materials and methods

### Bacterial strains, cell culture and chemicals

*E*. *coli* DH5α strain was used for cloning, *E*. *coli* BL21(DE3) or NiCo21(DE3) were used for protein expression (New England Biolabs). Bacteria were grown in LB-Miller medium, supplemented with antibiotic (ampicillin 100 μg/mL or kanamycin 30 μg/mL) when necessary. HeLa cells (HeLa-S3 cells, ATCC) were cultured in Dulbecco’s Modified Eagle Medium (DMEM, Life Technologies), supplemented with 10% heat-inactivated fetal bovine serum (FBS, Gibco) and 1% antibiotics (penicillin/streptomycin), at 37°C in a humidified atmosphere containing 5% CO2, and subcultured approximately every 2–3 days. All chemicals and reagents were purchased from Sigma.

### Plasmids, cloning and sequence analyses

The pET16b prokaryotic expression vector, coding for EcolCdtB-I, WT or H153A 10His-tagged, was used. pRSF-Duet1 plasmids coding for HducCdtB, wt or D273R 6His-tagged at their N-terminal part, were provided by Dr Frisan (Karolinska Institute, Stockholm, Sweden). All subunits were subcloned in frame with mCherry fluorescent protein, at XhoI and EcoRI sites of pmCherry-C1 vector. The following primers were used: HducCdtB (forward: 5’- TATATACTCGAGCAAACTTGAGTGACTTCAAAGT-3’, reverse: 5’- TATATAGAATTCTTAGCGATCACGAACAAAACT-3’) and EcolCdtB-I (forward: 5’-TATATACTCGAGCAGATTTAAGCGATTTTAAAGT-3’, reverse: 5’- TATATAGAATTCTCATCTTCTTGCTCCTCTTC-3’). Primers synthesis was performed by Sigma. Phusion polymerase and Restriction enzymes (FastDigest) used in molecular cloning experiments were from Thermo Scientific. PCR products were purified with GFXTM PCR DNA and gel band kit (Illustra-GE Healthcare). Ligation reactions were performed with Instant Sticky-end Ligase Master Mix (New England Biolabs) and transformed into DH5α strain. All constructs were analysed by DNA sequencing (Eurofins Scientific). CdtB and bovine DNase I sequences were retrieved from the NCBI Protein database and the multiple sequence alignment was performed with ESPript 3 [27].

### CDT subunits purification

EcolCDT holotoxin was purified as previously described [27]. For all other proteins, overnight bacterial cultures were grown in LB-Miller medium (14 h at 37°C, 250 rpm) supplemented with adequate antibiotic (ampicillin 100 μg/mL or kanamycin 30 μg/mL), then diluted in 1 L and incubated to reach 0.6 at OD600nm for native condition or 1 at OD600nm for denaturing purification. Protein expression was induced with 0.5 mM Isopropyl-β-D-thiogalactoside (IPTG) and incubated 12 h at 16°C, 250 rpm for native condition or induced with 0.1 mM IPTG and induced 3 h at 37°C, 250 rpm for denaturing purification. Cells were collected by centrifugation (4°C, 6000 g, 15 min) and pellets stored at -80°C.

For purifications under native conditions, each pellet was resuspended on ice in 10 mL of T5 buffer (Tris-HCl 40 mM pH 8, NaCl 500 mM, Glycerol 10%, Imidazole 5 mM, Lysozyme 1 mg/mL, Halt^TM^ Protease-and Phosphatase Inhibitor Cocktail 1x from Thermo Scientific). Lysis was performed by dounce-sonication cycles on ice (10 dounce cycle with the tight Potter B, followed by 10 sec of sonication pulse/30 sec pause during 10 minutes). Total extract was centrifuged (4°C, 20 000 g, 40 min) and supernatant (soluble extract) processed for further purification. Chitin Magnetic Beads (New England Biolabs) were used to remove CBD (Chitin Binding Domain)-tagged contaminants when using NiCo21 (DE3) strain. Flow-through was subjected to affinity purification on NiNTA beads (TALON Metal Affinity Resin, Clontech). One mL of dried NiNTA beads were prepared following the manufacturer’s instructions and incubated with the protein extract (4°C, 2 h, 10 rpm). After centrifugation (4°C, 700 g, 1 min), 150 μL of supernatant were kept (Flow-through). Beads were washed three times with 5 mL of T80 buffer (Tris-HCl 40 mM pH 8, NaCl 500 mM, Glycerol 10%, Imidazole 80 mM) at 4°C for 5 min, and centrifugated (4°C, 700 g, 1 min). First elution was performed with 6 mL of T500 buffer (Tris-HCl 40 mM pH 8, NaCl 500 mM, Glycerol 10%, Imidazole 500 mM), at 4°C during 20 min, followed by a second elution performed with 3 mL of T500 buffer. The eluted fractions were pooled before the next dialysis step, protein concentration was evaluated by Bradford assay (Bio-Rad) and protein samples were analysed by SDS-PAGE.

For purifications under denaturing conditions, each pellet was resuspended on ice in 8 mL of lysis buffer (Tris-HCl 0.05 M pH 7.5, Sucrose 0.75 M, EDTA 500 mM, lysozyme 1x, PMSF 0.1 mM) and incubated 30 min. Lysis was performed by dounce-sonication cycles (15 dounce with tight Potter B, followed by 30 sec of sonication pulse/30 sec pause on ice during 10 minutes), and 16 mL of Detergent Buffer (Tris-HCl 0.02 M pH 7.5, NaCl 0.18 M, Sodium Deoxycholate 1%, Igepal 25 mM, EDTA 2 mM, 2-β-ME 0.07%) were added. After centrifugation (4°C, 15 000 g, 30 min), pellets were resuspended in 4 mL of Tris-HCl 0.02 M pH 7.5, Triton X-100 0.5%, EDTA 1 mM, 2-β-ME 0.07%, sonicated 10 sec and centrifuged (4°C, 14 000 g, 10 min). These sonication/centrifugation steps were repeated until pellet had a white color. Triton X-100 was then removed by pellet resuspension in 4 mL of Tris-HCl 0.05 M pH 7.5, EDTA 1 mM, DTT 10 mM. After centrifugation (4°C, 14 000 g, 10 min), pellet was resuspended in 8 mL of Binding Buffer (Urea 7 M, NaCl 0.5 M, Sodium Phosphate 20 mM pH 7.2, Imidazole 10 mM), incubated 20 min on ice to lyse inclusion bodies, and centrifuged (4°C, 14000 g 10 min). Supernatant was then incubated with Cobalt beads (HisPur Cobalt Resin, ThermoScientific) (4 mL of dried and washed beads), for 1 h at 4°C. After centrifugation (4°C, 700 g, 5 min), flow-through was removed and beads were washed two times (4°C, 10 min at 10 rpm) with Q.S. 15 mL of Binding Buffer. Proteins were eluted four times (4°C, 10 min at 10 rpm) with buffers containing increasing concentrations of imidazole (1^st^, 2^nd^ and 3^rd^ elution with Q.S. 15 mL of Elution Buffer 60 mM (Urea 7 M, NaCl 0.5 M, Sodium Phosphate 20 mM pH 7.2, Imidazole 60 mM); 4^th^ wash with Buffer 300 mM (Urea 7 M, NaCl 0.5 M, Sodium Phosphate 20 mM pH 7.2, Imidazole 300 mM)). Protein refolding was performed by dialysis.

### Dialysis and HducCDT reconstitution

For dialysis, 10 kDa cut-off Dialysis Cassettes (Slide-A-Lyzer, ThermoScientific) were used. For proteins eluted in native conditions, dialysis was done with (Tris-HCl 20 mM pH 8, Glycerol 10%, Tween 0.05%, NaCl 250 mM, DTT 0.5 mM) at 4°C. After 1 h, buffer was changed and dialysis performed overnight (12 h). For proteins eluted in denaturing conditions, seven successive dialysis steps were performed at 4°C, with 10 kDa cut-off Dialysis Cassettes (Slide-A-Lyzer, ThermoScientific). HducCDTs were reconstituted by co-dialysis at equimolar concentrations. The first four baths used Refolding Buffer with decreasing Urea and L-Arginine concentration (Tris-HCl 100 mM pH 7.5, L-Arginine 400 mM, Glycerol 20%, Urea 5 M or 3 M or 1.5 M for baths 1 to 3, Tris-HCl 100 mM pH 7.5, L-Arginine 200 mM, Glycerol 20% for bath 4) and were changed after 24 h. The proteins were then dialyzed three times with Tris-HCl 100 mM pH 7.5, Glycerol 10% during 3 h. Finally, the protein solution was centrifuged (4°C, 13 000 g, 5 min) to remove any aggregate, snap-frozen and stored (-80°C).

### SDS-Polyacrylamide Gel Electrophoresis (PAGE) and Western blots

Quantity and purity of CdtB subunits were determined by SDS-PAGE and Coomassie staining or Stain Free technology (TGX FastCast Acrylamide gel, BioRad), using bovine β-casein as standard. Samples were boiled in Laemmli and separated by SDS-PAGE. Coomassie staining was performed with PageBlue Protein Staining Solution (ThermoScientific). For Western blots experiments, proteins were transferred on nitrocellulose membrane (Amersham) and blocked in Tris-HCl 20 mM pH 7, NaCl 150 mM (TBS 1x) 50/50 v/v with Odyssey Blocking Buffer (Rockland), Tween 20 0.5% sodium azide 0.02% for 1 h, then incubated for 1 h with monoclonal anti-Histidine (Clontech), washed three times in TBS 1X Tween 20 0.05% and incubated 45 min with a fluorescent goat anti-mouse secondary antibody (CF770, Biotium). Immunoblots were revealed with an Odyssey Infrared Imaging Scanner (Li-Cor ScienceTec).

### Size Exclusion Chromatography (SEC) and Dynamic Light Scattering (DLS)

SEC experiments have been performed using a Superdex 75 HR10/300 analytic column (22 mL, exclusion limit of 70 kDa) with an AKTA Purifier10 chromatographic system (GE Healthcare, France). The detection was done by absorbance measurement at 215nm, 254nm and 280nm. Elution was performed at a flow rate of 0.450 mL/min with Tris-HCl 100 mM, NaCl 100 mM, glycerol 10%, pH 7.5 or TrisHCl 20 mM, NaCl 250 mM, glycerol 10%, pH 8 for proteins purified in denaturing or native conditions, respectively. Fractions of 250 μL were collected for analysis. Finally, Dynamic Light Scattering analysis has been performed using a NanoStar instrument (Wyatt Technology, France).

### Nano liquid chromatography-mass spectrometry analysis

Samples were prepared for mass-spectrometry analyses as previously described [28]. Briefly, eluted proteins were precipitated with 0.1 M ammonium acetate in 100% methanol. After a reduction-alkylation step (Dithiothreitol 5 mM—Iodoacetamide 10 mM), proteins were digested overnight with 1/25 (W/W) of modified sequencing-grade trypsin (Promega) in 50 mM ammonium bicarbonate. Resulting peptides were vacuum-dried in a SpeedVac concentrator and re-suspended in water containing 0.1% Formic Acid (solvent A) before being injected on nanoLC-MS/MS (NanoLC-2DPlus system with nanoFlexChiP module (Eksigent, ABSciex, Concord, Canada), coupled to a TripleTOF 5600 mass spectrometer (ABSciex)). The mass spectrometry instrumentation was granted from Investissement d’Avenir program (NetRNA ANR-10-LABX-36).

Peptides were eluted from C-18 analytical column (75 μm ID x 15 cm ChromXP; Eksigent) with 1 h—or 2 h-long gradients of acetonitrile (solvent B) depending on sample concentration. Data were compared with the complete *E*. *coli* proteome set from SwissProt database (released 2013/01/09; 4 303 sequences). Peptides were identified with Mascot algorithm (version 2.2, Matrix Science, London, UK) through the ProteinScape 3.1 package (Bruker). Identified proteins were validated with a minimum score of 30, a p-value<0.05, a minimum of 2 identified peptides and a decoy database strategy was employed to validate Mascot identifications at FDR < 1%. Classic contaminants as keratins and carry-over from previous experiments were removed (no topoisomerase or any enzymatic contaminant were found).

### Plasmid digestion activity assay

To assess Hduc- or EcolCdtB nuclease activity *in vitro*, 250 ng or 125 ng of the supercoiled plasmid pRSET-A (Invitrogen) resuspended in water was incubated at 37°C with 9 ng to 1 μg of the CdtB subunit, for 30 min to 7 h, in digestion buffer (20 mM TrisHCl pH 7.5, 50 mM NaCl, 5 mM CaCl2, 5 mM MgCl2, 50 μg/mL BSA), containing 10 mM of divalent cation as recommended previously [18]. The reaction was stopped by adding 10 mM of EDTA (final concentration) and Gel Loading Dye blue (1x final concentration, New England Biolabs). The digestion products were analysed on 1% agarose gel (migration 25 min at 75 V), stained by ethidium bromide. Plasmid was incubated at 37°C in absence of CdtB subunit or in presence of HducCdtC for negative control, or in presence of recombinant bovine DNase I (2000 u/mL, New England Biolabs) for positive control. The band intensities of supercoiled, relaxed and linear plasmids were quantified with ImageJ software.

### Oligonucleotide digestion activity assay

A 60-mer oligonucleotide (sequence: GGGTGAACCTGCAGGTGGGCAAAGATGTCCTAGCAATGTAATCGTCAAGCTTTATGCCGT) fluorescently-labbeled with CF^TM^680 in 5’ (Sigma) was hybridized or not with its complementary strand to form the dsDNA and ssDNA substrates, respectively. dsDNA substrates were separated from unlabelled oligonucleotides by electrophoretic migration on 8% polyacrylamide gel in TAE. DNA from the band containing the dsDNA substrate was electro-eluted in water with GeBAflex tubes (Euromedex). The dsDNA substrate concentration was estimated on 8% polyacrylamide gel from the ssDNA substrate. 100 nM of dsDNA or ssDNA was incubated for with 50 ng of CdtB for 2 h at 37°C in in digestion buffer (20 mM TrisHCl pH 7.5, 50 mM NaCl, 50 μg/mL BSA), containing or not 10 mM of divalent cation (5 mM CaCl2 and 5 mM MgCl2). Bovine DNase I (New England Biolabs) was used as positive control. The reaction was stopped by a 15 min incubation with 2 mg/mL of proteinase K and 2% SDS. The digestion products were separated on 8% polyacrylamide gel in TAE (migration 30 min at 150 V) and analysed using an Odyssey Infrared Imaging Scanner (Li-Cor ScienceTec).

### Cell culture, transfection and immunofluorescence

For plasmid transfection, 100,000 HeLa cells cells were seeded in 6-well plates on glass coverslips. Plasmid transfection was performed with TransIT-2020 (MiriusBio) according to manufacturer’s instructions for 8 h, 9.5 h or 11 h. For protein transfection, 40,000 HeLa cells were seeded in 24-well plates on glass coverslips. Protein transfection was performed with TransIT-X2 (MiriusBio) according to manufacturer’s instructions at different protein concentrations and for different incubation time (see figures for details).

For immunofluorescence analyses, cells were fixed in 4% paraformaldehyde and washed once in Phosphate Buffered Saline (PBS), before permeabilization with PBS Triton X-100 (0.5%) for 15 min. Cells were washed once in washing buffer (PBS, NP40 0.1%) and blocked 40 min in washing buffer supplemented with BSA 3%. Cells were incubated with the mouse anti-γH2AX antibody (Epitomics), and, when specified, with the rabbit anti-53BP1 antibody (Novus Biologicals) diluted 1/1,000 in blocking buffer for 1 h, washed three times in washing buffer, and incubated with the Alexa 488-conjugated goat anti-mouse and, for 53BP1 co-immunostaining, the Alexa 546-conjugated goat anti-rabbit secondary antibodies (Molecular Probes, Sigma), diluted 1/800 in washing buffer for 1 h. Nuclei were counterstained with DAPI, and cells were washed three times 10 min in washing buffer and mounted in p-Phenylenediamine (PDA) (Sigma-Aldrich). Analyses were performed with fluorescent microscope (Nikon, Eclipse 50i), objective 40x. For plasmid transfection experiments, cells presenting visible mCherry signal were considered high-expression level cells, whereas those presenting mCherry signal visible only after image treatment with ImageJ were considered as low-expression cells; cells presenting a green pan-nuclear staining were considered positive for γH2AX staining. For holotoxin treatment and protein transfection experiments, the number of DAPI-positive cells and the intensity of γH2AX in nuclei were determined with ImageJ software. For each experiment, 100 to 200 cells were counted, and at least 3 independent experiments were performed.

### Data analysis

The results are expressed as the mean ± SD of at least 3 independent experiments. Statistical analysis was assessed using Prism 4 software (GraphPad Software Inc., San Diego, CA). Two-way ANOVA followed by Sidak’s multiple comparison tests were used in order to study treatment effects and HducCDT vs EcolCDT effect. The differences were considered significant at P < 0.05.

## Results

**Fig 1:**
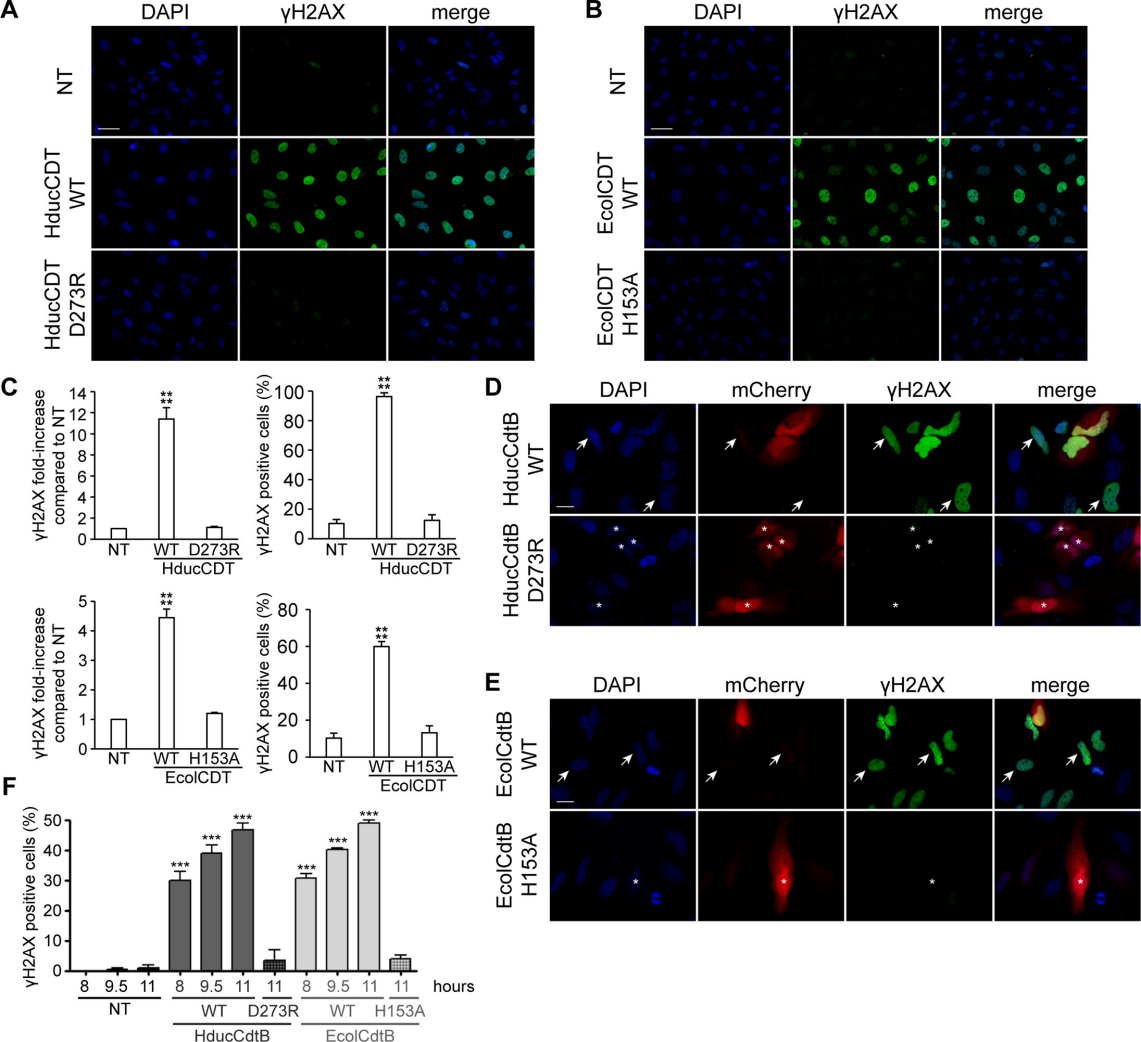
DNA damage induction in HeLa cells after CDT holotoxin treatment or mCherry-CdtB expression. A. Representative images of γH2AX immunofluorescence and DAPI staining from HeLa cells treated with 35 ng/mL of WT or D273R HducCDT holotoxin for 24 h. Scale bar: 50 μm. B. Representative images of γH2AX immunofluorescence and DAPI staining from HeLa cells treated with 2.5 ng/mL of WT or H153A EcolCDT holotoxin for 24 h. Scale bar: 50 μm. C. Quantification of γH2AX signal in HeLa cells left untreated (NT), treated with 20 ng/mL of WT or D273R HducCDT or with 2.5 ng/mL of WT or H153A EcolCDT for 24 h, represented as the mean fluorescence intensity per cell (normalised to 1 for the untreated condition) or as the proportion of γH2AX positive cells. Results present the mean ± SD of at least three independent experiments; statistical differences were analysed between treated and untreated conditions (**** P < 0.0001). D. Representative images of mCherry-HducCdtB localisation, γH2AX immunofluorescence and DAPI staining from HeLa cells expressing WT or D273R HducCdtB in fusion with mCherry. Immunostaining was performed 11 h after transfection. Cells with high expression (stars) or low expression (arrows) of WT mCherry-HducCdtB are indicated. Scale bar: 20 μm. E. Representative images of mCherry-EcolCdtB localisation, γH2AX immunofluorescence and DAPI staining from HeLa cells expressing WT or H153A EcolCdtB in fusion with mCherry. Immunostaining was performed 11 h after transfection. Cells with high expression (stars) or low expression (arrows) of WT mCherry-EcolCdtB are indicated. Scale bar: 20 μm. F. Quantification of γH2AX positive HeLa cells left untransfected (NT), expressing WT or mutant (HducCdtB D273R or EcolCdtB H153A) CdtB in fusion with mCherry. Immunostaining was performed at the indicated time after transfection. Results present the mean ± SD of at least three independent experiments; statistical differences were analysed between transfected and non-transfected conditions at each time point (*** P < 0.001).

CDT is known to induce DNA damage in mammalian cells that can be monitored through H2AX phosphorylation on S139 [26] referred to as γH2AX. Hence, HeLa cells cultivated during 24 hours in presence of 2.5 ng/mL of CDT from HducCDT, exhibit a strong γH2AX signal in immunofluorescence analyses, compared to control cells (Fig 1A). In contrast, the D273R mutant, a residue previously shown to be essential for EcolCdtB activity (S1 Fig and [12]) did not increase the level of γH2AX staining compared to the background observed in the non-treated cells, confirming that D273 is required for HducCDT activity. The same experiment was conducted with EcolCDT, using the WT or the H153A catalytically inactivating mutation [27]. While WT EcolCDT generates a strong H2AX phosphorylation in almost all exposed cells, the H153A mutant did not induce DNA damage. Next, we verified that the lack of DNA damage observed with CdtB mutants is not a consequence of a defective holotoxin formation or CdtB transport to the nucleus. CdtB cDNA from Hduc and Ecol were sub-cloned into mammalian expression plasmids, in fusion with mCherry (pmCherry). HeLa cells were transiently transfected with pmCherry-HducCdtB WT or D273R plasmids (Fig 1D) and with pmCherry-EcolCdtB WT or H153A plasmids (Fig 1E). HeLa cells were transfected for 11 hours before fixation for subsequent γH2AX immunostaining (Fig 1D and 1E). Consistently with the holotoxin treatment, WT HducCdtB and EcolCdtB induced DNA damage while the inactive mutants did not, even after a longer transfection time of 24 hours were DNA damage-related cell death is already visible (S2 Fig). WT CdtB-mediated γH2AX induction was observed even in cells with barely detectable mCherry signal (depicted by arrows in Fig 1D and 1E), indicating that even extremely low expression levels of CdtB are sufficient to generate DNA damage. In contrast, cells with high fluorescence intensity of the mutant mCherry-CdtB were negative for γH2AX (stars in Fig 1D and 1E). Both WT HducCdtB and EcolCdtB direct expression increased the proportion of γH2AX-positive cells in the entire cell population, in a time dependent-manner (Fig 1F). In order to show that these observations are not restricted to a specific cell line, the same experiments of holotoxin exposure and pmCherry-CdtB transfection were also performed with the human osteosarcoma U2OS cells, and similar results were obtained (S3 Fig). Taken together, these results confirm that DNA damage observed through γH2AX induction allows discriminating inactive mutants from WT CdtB after direct expression in living cells.

**Fig 2:**
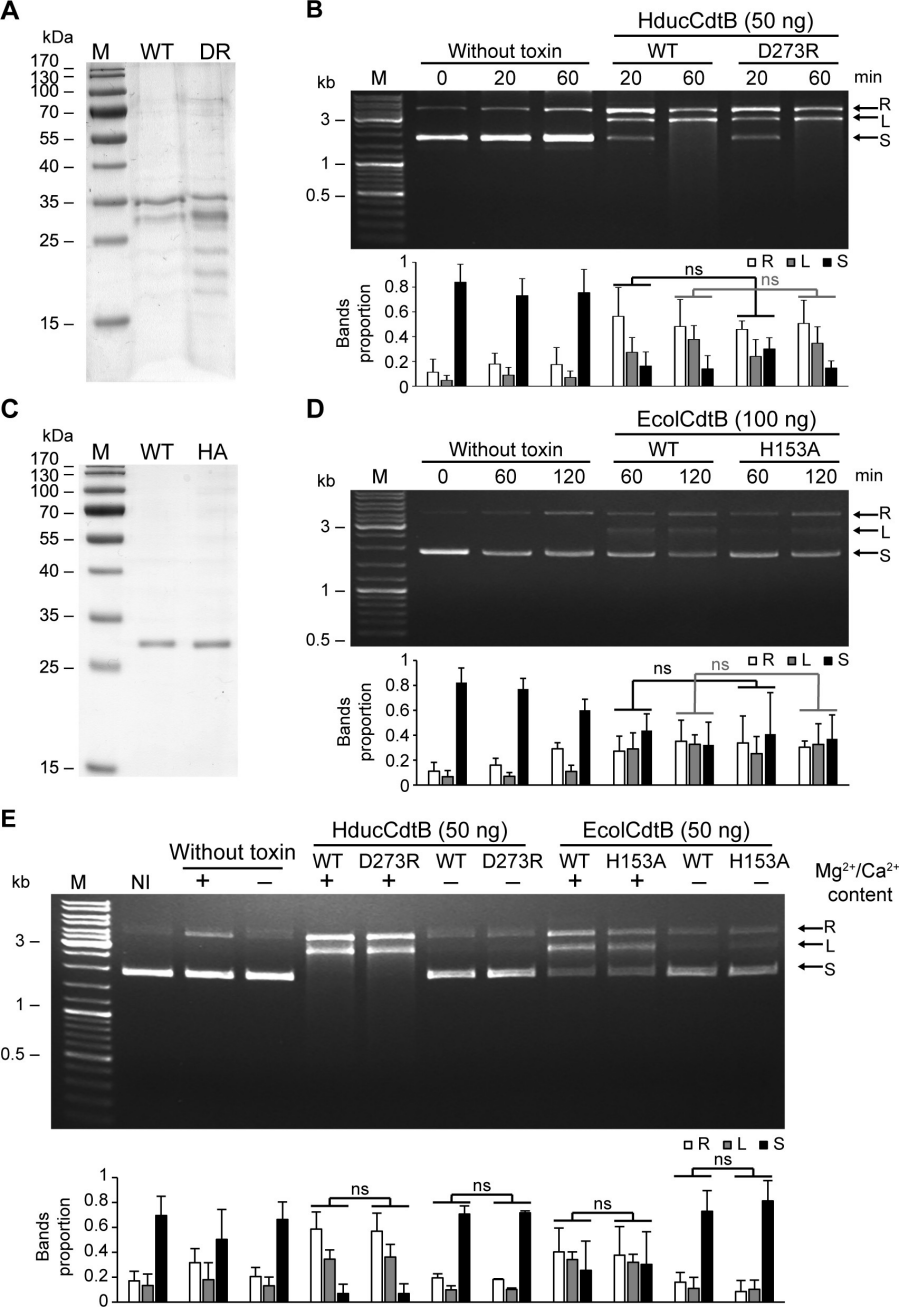
Wild-type or catalytic mutant of EcolCdtB and HducCdtB purified under native condition present a similar plasmid digestion activity. A. SDS-PAGE analysis of WT and D273R (DR) HducCdtB proteins purified from *E*. *coli* NiCo21 (DE3) under native conditions. M: molecular weight marker. B. Kinetics of the plasmid digestion assay in presence of WT or D273R HducCdtB. Agarose gel electrophoresis and quantification of supercoiled plasmid (250 ng) incubated with or without 50 ng of WT or D273R HducCdtB for the indicated time. M: molecular weight marker. C. SDS-PAGE analysis of WT and H153A (HA) EcolCdtB proteins purified from *E*. *coli* NiCo21 (DE3) under native conditions. M: molecular weight marker. D. Kinetics of the plasmid digestion assay in presence of WT or H153A EcolCdtB. Agarose gel electrophoresis and quantification of supercoiled plasmid (250 ng) incubated with or without 100 ng of WT or H153A EcolCdtB for the indicated times. M: molecular weight marker. E. Ions content effects on plasmid digestion by CdtB. Agarose gel electrophoresis and quantification of supercoiled plasmid (250 ng) incubated in presence or absence of 50 ng of WT or mutant (Hduc D273R or Ecol H153A) CdtB for 2 h, in presence or absence of Mg2+/Ca2+. M: molecular weight marker. B, D and E: arrows indicate plasmid conformation, either relaxed (R), linear (L) or supercoiled (S). For quantifications, the amount of each plasmid conformation is expressed as proportion of the total plasmid content. Results present the mean ± SD of at least three independent experiments; statistical differences were analysed between every conditions and only mutant vs WT comparisons are shown (ns: not significant).

To confirm loss of nuclease activity in CdtB mutants, classical biochemical DNase assays were performed. Recombinant HducCdtB was produced in NiCo21 (DE3) *E*. *coli* and purified under native conditions (Fig 2A). The purified subunits were then incubated with a plasmid preparation during 20 or 60 minutes and plasmid degradation was assessed by migration on agarose gel electrophoresis (Fig 2B). We observed an increase of the relaxed and supercoiled forms of the plasmid from 20 minutes of incubation with the WT or the mutant HducCdtB, in a similar range. Comparable results were obtained from WT and H153A EcolCdtB purified under native conditions (Fig 2C and 2D). In the same way, dose-response analysis did not allow to differentiate HducCdtB or EcolCdtB nuclease activity from their mutant counterparts (S4 Fig). These results indicate the presence of nuclease activity in all CdtB preparations, independently of the mutation shown to inhibit DNA damage formation in cellular assays (Fig 1). As the catalytic activity of CdtB was shown to depend on Mg^2+^ and Ca^2+^ [18], plasmid digestion was assessed during 2 hours in presence or absence of divalent cations (Fig 2E). Strikingly, a part of relaxed plasmid formation during incubation is only due to the presence of ions, as observed in absence of CdtB. The presence of Mg^2+^ and Ca^2+^ greatly increases plasmid degradation for both WT and mutants CdtB. Yet, some nuclease activity is still visible in absence of Mg^2+^ and Ca^2+^ cations. Whatever the condition, the proportion of degraded plasmid cannot be distinguished between WT and mutant CdtB. Thus, in contrast to cellular experiments, recombinant WT CdtB and catalytic mutants purified under native conditions display an analogous capacity to induce plasmid degradation, with part of the nuclease activity being independent of divalent cations.

**Fig 3:**
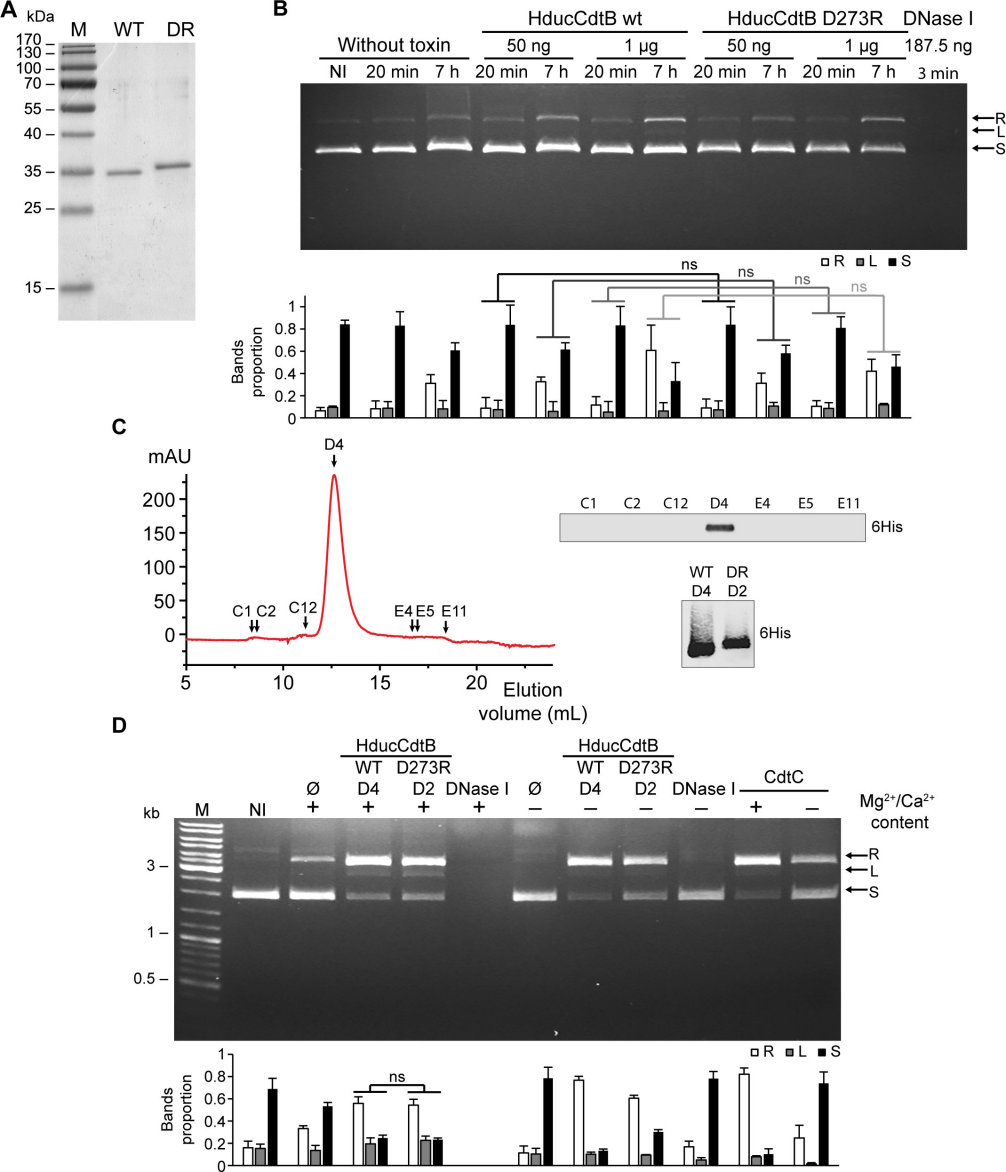
Wild-type or mutant HducCdtB proteins purified under denaturing conditions present a similar plasmid digestion activity. A. SDS-PAGE analysis of WT and D273R (DR) HducCdtB proteins purified from *E*. *coli* NiCo21 (DE3) under denaturing conditions. B. Kinetics and CdtB concentration effect on plasmid digestion assay with WT or D273R HducCdtB. Agarose gel electrophoresis and quantification of supercoiled plasmid (250 ng) incubated in presence of the indicated concentration of WT or D273R HducCdtB or bovine DNase I for the indicated times. C. Size exclusion chromatography (SEC) of WT HducCdtB. The curve represents the absorbance at 280 nm of the fractions eluted from the SEC column. The fractions used for further analysis are shown with black arrows. The selected fractions were analysed by Western Blot with anti-HIS antibody. The peak fractions from WT and D273R (DR) HducCdtB were analysed by Western Blot with anti-HIS antibody. D. Plasmid digestion assay with WT or D273R HducCdtB purified from SEC. Agarose gel electrophoresis and quantification of supercoiled plasmid (125 ng) incubated in presence of 1 μg of WT (D4) or D273R (D2) HducCdtB or in control conditions (no protein, with 2 ng of bovine DNase I or with 1 μg of CdtC) for 7 h in presence or in absence of Mg2+/Ca2+ buffer. B and D: arrows indicate plasmid conformation, either relaxed (R), linear (L) or supercoiled (S). For quantifications, the amount of each plasmid conformation is expressed as proportion of the total plasmid content. Results present the mean ± SD of at least three independent experiments, except for the D4 and D2 fractions without ions which were replicated twice; statistical differences were analysed between every conditions and only mutant vs WT comparisons are shown (ns: not significant).

The apparent DNase activity of the CdtB fractions could be due to the presence of a contaminant. To rule out this hypothesis, we performed a more stringent purification of HducCdtB under denaturing conditions (Fig 3A). Plasmid preparation was then incubated with 50 ng or 1μg of WT or D273R HducCdtB during 20 minutes or 7 hours (Fig 3B). The nuclease activity of the bovine DNase I was assessed as a positive control and showed a complete degradation of the plasmid in only 3 minutes. In contrast, a very limited nuclease activity was observed for HducCdtB fractions. As with CdtB purified under native conditions, WT and D273R HducCdtB displayed similar capacities to induce plasmid degradation, independently of the toxin concentration and incubation time. The HducCdtB proteins were then further purified by size exclusion chromatography (SEC), and the presence of HducCdtB in the fractions corresponding to the protein peak was confirmed by western-blot (Fig 3C and S5 Fig). The purity of WT (fraction D4) and D273R (fraction D2) HducCdtB was verified by mass spectrometry (S6 Fig). The nuclease activity of both SEC fractions was evaluated by plasmid digestion assay. As observed before SEC, WT and D273R HducCdtB exhibit the same nuclease activity (Fig 3D). Again, the absence of ions does not abolish HducCdtB-mediated plasmid degradation, contrary to DNase I. Interestingly, HducCdtC, devoid of any nuclease activity [12] is also able to induce plasmid digestion, with or without divalent cation. In conclusion, the plasmid degradation observed in this assay, with either WT or catalytically inactivated HducCdtB or with HducCdtC, may not be due to the activity of a functional nuclease domain. Based on these experiments, the classic plasmid digestion assay is therefore not relevant to study CdtB nuclease activity.

In addition, we also asked whether linear double-stranded (dsDNA) or single-stranded DNA (ssDNA) could constitute a better substrate to test CdtB nuclease activity. A fluorescently-tagged 60-mer oligonucleotide, hybridized or not to its complementary strand, was used to form the dsDNA and ssDNA linear substrates, respectively (S7A Fig). Both substrates were completely degraded by DNase I. Incubation with WT but also catalytic mutants of HducCdtB and EcolCdtB purified under native conditions induced some dsDNA and ssDNA degradation (S7B and S7C Fig). This activity was strongly diminished in absence of divalent cations, and totally abolished when HducCdtB was purified under denaturing conditions (S7D Fig). Thus, this assay does not distinguish the WT construct from CdtB mutants, indicating that similarly to the plasmid digestion assay, the substrate degradation is independent on CdtB catalytic activity.

**Fig 4:**
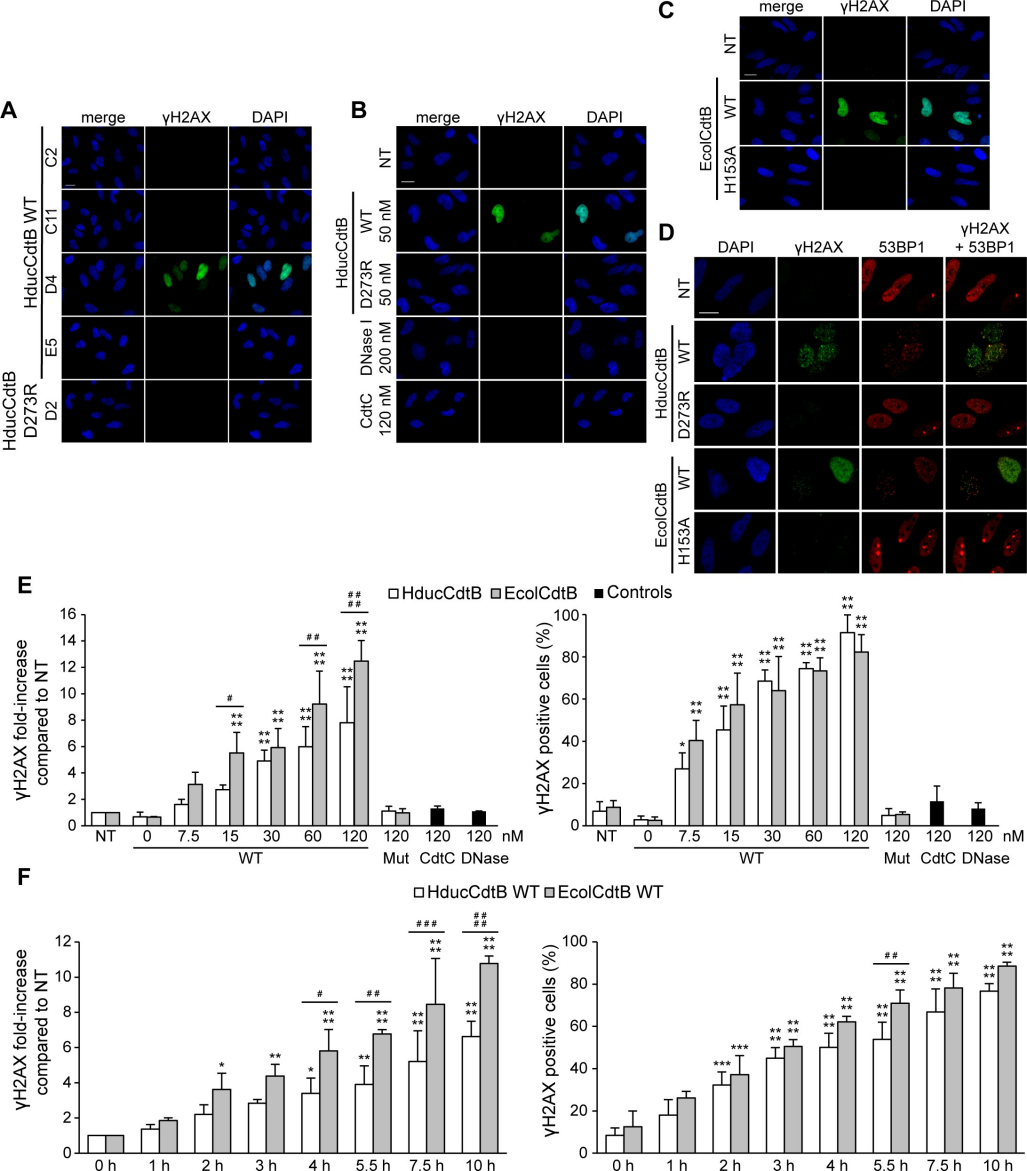
DNA damage induction in HeLa cells transfected with purified CdtB. A. Representative images of γH2AX immunofluorescence and DAPI staining from HeLa cells transfected with 50 nM of HducCdtB WT (fractions C2, C11, D4, E5) or D273R (fraction D2) after SEC for 24 h. Scale bar: 20 μm. B. Representative images of γH2AX immunofluorescence and DAPI staining from HeLa cells transfected with 50 nM of WT or D273R HducCdtB before SEC, with 200 nM of DNase I or with 120 nM of HducCdtC for 24 h. Scale bar: 20 μm. C. Representative images of γH2AX immunofluorescence and DAPI staining from HeLa cells transfected with 120 nM of EcolCdtB WT or H153A for 14 h. Scale bar: 20 μm. D. Representative images of γH2AX and 53BP1 immunofluorescence and DAPI staining from HeLa cells transfected with 5 nM of WT or mutant (Hduc D273R or Ecol H153A) CdtB for 14 h. Scale bar: 20 μm. E. Dose-response analysis of γH2AX induction after CdtB transfection. Quantification of γH2AX signal in HeLa cells left untransfected (NT) or transfected with the indicated concentration of WT or mutant (Hduc D273R or Ecol H153A) CdtB or negative controls (HducCdtC or DNase I) for 14 h, represented as the mean fluorescence intensity per cell (normalised to 1 for the untreated condition) or as the proportion of γH2AX positive cells. Results present the mean ± SD of at least three independent experiments. Statistical differences were analysed between transfected and non-transfected conditions for each CdtB concentration (* P < 0.05; **** P < 0.0001) or between HducCdtB and EcolCdtB (# P < 0.05; ## P < 0.01; #### P < 0.0001). F. Kinetics of γH2AX induction after CdtB transfection. Quantification of γH2AX signal in HeLa cells left untransfected (NT) or transfected with 120 nM of WT EcolCdtB or HducCdtB for the indicated time, represented as the mean fluorescence intensity per cell (normalised to 1 for the untreated condition) or as the proportion of γH2AX positive cells. Results present the mean ± SD of at least three independent experiments; statistical differences were analysed between transfected and non-transfected conditions for each time point (* P < 0.05; ** P < 0.01; ***P < 0.001; **** P < 0.0001) or between HducCdtB and EcolCdtB (# P < 0.05; ## P < 0.01; ### P < 0.001; #### P < 0.0001).

In order to validate the ability of purified CdtB to induce DNA damage, a novel cellular strategy was considered. HeLa cells were transfected for 24 hours with different SEC fractions from the HducCdtB WT purification (Fig 4A). Only the WT HducCdtB-positive D4 fraction increased γH2AX level. In contrast, the D273R HducCdtB-positive fraction D2 did not induce any DNA damage. The same experiment was conducted with HducCdtB purified under denaturing conditions before SEC, with DNase I or with HducCdtC serving as negative control (Fig 4B). Similarly to HducCdtC, DNase I did not induce γH2AX signal in transfected cells, possibly due to incapacity to enter the nucleus. WT HducCdtB generated a strong H2AX phosphorylation signal whereas D273R HducCdtB did not. HeLa cells were also transfected with WT and H153A EcolCdtB purified under native conditions (Fig 4C). While WT EcolCdtB greatly increased the γH2AX level in transfected cells, the inactive EcolCdtB mutant did not. A more careful observation shows that the WT CdtB-induced γH2AX increase does not reflect the pan nuclear staining observed during the apoptotic cascade, but rather forms nuclear foci that colocalise with 53BP1, a protein specifically recruited to DNA double-strand breaks [28] (Fig 4D). Taken together, these results show a clear difference between the genotoxic activity of purified WT and inactive CdtB mutants in a cellular assay, whereas these same purified toxins exhibited similar nuclease activity on classic plasmid digestion assay. Finally, to determine whether this cellular assay could be quantitative, we performed time-course and dose-response analyses. HeLa cells were transfected with 7.5 to 120 nM of WT EcolCdtB or HducCdtB, 120 nM of H153A EcolCdtB or D273R HducCdtB for 14 hours (Fig 4E). Both γH2AX signal intensity and proportion of γH2AX-positive cells increased with the concentration of transfected WT CdtB, while the γH2AX signals observed from cells transfected with mutant CdtB subunits were not significantly different from the untreated cells. Interestingly, EcolCdtB induced significantly more γH2AX signal than HducCdtB, whereas the proportion of γH2AX-positive cells was similar, indicating that EcolCdtB showed a greater genotoxic activity in HeLa cells compared to HducCdtB. Similar data was obtained in U2OS cells in which EcolCdtB induces more γH2AX signal compared to HducCdtB (S3E and S3F Fig). Then, HeLa cells were transfected with 120 nM of EcolCdtB or HducCdtB WT for 0 to 10 h (Fig 4F). The fluorescence intensity and proportion of damaged cells also increased with the transfection duration. In contrast, the catalytic mutants never increased γH2AX staining, even after 48 hours of transfection (S8 Fig). As shown in Fig 4D, EcolCdtB seemed more active than HducCdtB. Thus, these data showed a correlation between the signal intensity and proportion of γH2AX-positive cells and both the CdtB concentration and transfection duration, therefore confirming the quantitative aspect of this cellular CdtB activity assay. In conclusion, the proposed cellular assay is relevant to study and compare the nuclease activity of different CdtB subunits.

## Discussion

The classic plasmid digestion assay developed to study DNase I activity [29] has been transposed to EcolCdtB nuclease activity testing on the basis of their phylogenetic relationships [12], and has since been widely used with many other CdtB subunits [10,17–20,22,24,30–32]. However, comparison of CdtBs and DNase I nuclease activities showed that CdtBs are 100 to 10.000-fold less active [17–19,31,32]. Under our conditions, less than 200 ng of DNase I completely degrade the DNA substrate in only 3 minutes, whereas up to 1 μg of CdtB can barely digest the plasmid over the course of several hours, i.e. resulting in the formation of relaxed and few linear molecules. This confirms that CdtB is far less active than DNase I, or that the plasmid digestion assay is not totally transferable to the CdtB study. In this study, we present evidence supporting that classic plasmid digestion assay cannot properly measure CdtB nuclease activity and propose an alternative cellular approach.

Several concerns prompted us to reconsider the relevance of the biochemical testing of CdtB nuclease activity by the plasmid digestion assay. Many studies reported a diminution of plasmid degradation caused by CdtB catalytic mutations, but with contrasting magnitude compared to DNase I activity. Indeed, the H160Q AactCdtB mutant shows a 4-fold drop in activity [22] while catalytic mutation in human DNase I decreased activity by up to 170 000 times [29]. Moreover, previous studies have reported contradictory results regarding the capacity of different CdtB mutants to digest plasmid DNA (see introduction), despite similar loss of cellular defects induced by these mutations in the context of holotoxin [10,12,22,23]. Here, when comparing WT and catalytically inactive CdtB, only WT holotoxin induced DNA damage on exposed cells whereas the plasmid digestion assay showed no difference between WT and mutant subunit activities. We could not exclude the hypothesis that the apparent nuclease activity was attributable to a contamination, especially when CdtB subunits were purified under native conditions. To rule out this possibility, CdtB fractions purified to near-homogeneity under denaturing conditions were subject to an additional purification step by SEC. Furthermore, analysis by mass spectrometry analyses did not reveal any contamination, strongly supporting that the plasmid digestion activity can only be attributable to CdtB. Whatever the purification strategy employed, we never observed any difference in plasmid degradation between WT and inactive mutant. Based on these data and the statement that high amount of CdtB and long incubation time are necessary to detect very limited nuclease activity, in comparison with DNase I or other deoxyribonucleases ([33] and references herein), we concluded that plasmid degradation after incubation with purified CdtB does not depend on its intrinsic catalytic activity and might therefore be considered artefactual.

To strengthen this assumption, we tested the dependence of CdtB nuclease activity on its theoretical cofactors. Based on the cation-interacting residues conservation from DNase I [11,12,17] and the overall similarities between the two proteins, CdtB has previously been described as a Ca2+ and Mg2+ dependant nuclease [18]. In contrast to DNase I, plasmid digestion activity of the CdtB SEC fractions did not depend on the presence of divalent cations, and thus did not depend on CdtB catalytic site. On the other hand, a small decrease in activity of CdtB purified under native conditions was observed without Ca2+ and Mg2+. Besides, the mere presence of cations during plasmid incubation at 37°C is sufficient to induce some degradation in the absence of protein, confirming the possible presence of contaminant nucleases in these fractions. Finally, unexpected plasmid degradation was detected with HducCdtC, which is devoid of any nuclease domain and do not induce any DNA damage when expressed or introduced in human cells [11,34]. Another group already mentioned similar unsuitable plasmid digestion activity with CdtC from Aact, but also with CdtA and a CdtB variant lacking the catalytic H160 residue [35]. Taken together, these data firmly question the relevance of the plasmid digestion assay in the analysis of CdtB nuclease activity, implying that other approaches must be considered.

Several cellular assays have already demonstrated CdtB ability to damage genomic DNA. Chromatin fragmentation after CDT exposure can be directly visualized by pulsed-field gel electrophoresis [36,37] or comet assay [27,32,38]. Another way to monitor CDT-mediated cellular DNA damage relies on the detection of specific biomarkers, the most commonly used being γH2AX, either by western-blot [19,32,34,39–41] or immunofluorescence experiments [7,19,27,32,38,41–43]. In this study, γH2AX immunodetection was performed in HeLa and U2OS cells after expressing the CdtB-mCherry construct or after transfecting the recombinant CdtB. Compared to undamaged mCherry-positive cells transfected with the plasmid harboring the CdtB catalytic mutant, cells expressing WT CdtB-mCherry presented a very strong pan-nuclear H2AX phosphorylation signal, revealing huge DNA damage, which is quite contrasting to the limited plasmid digestion activity observed *in vitro* with WT or mutated CdtB subunits. Nevertheless, one has to remember that the apoptotic cascade induces a peculiar pan-nuclear γH2AX signal due to global chromatin fragmentation [28]. In this study, cells were not transfected for more than 11 hours to avoid apoptotic DNA compaction observed at longer CdtB-mCherry expression time (S2 Fig), as described in a previous study [11]. Therefore, we cannot exclude that the γH2AX staining induced by CdtB-mCherry could result from an apoptotic process rather than a direct response to DNA damage. Indeed, apoptosis-dependent DNA fragmentation has been documented in response to CDT holotoxin, but more specifically in lymphoid cell lines where the lipid phosphatase rather than the nuclease activity of CdtB has been shown to mediate CDT cytotoxicity [6,23,44,45]. In contrast, the important γH2AX staining induced by the direct expression of CdtB in HeLa cells more probably results from rapid and massive DNA lesions. Indeed, the DNA damage-independent apoptotic process is characterised by a rapid transition from the γH2AX pan-nuclear staining to DNA loss and compaction [28]. Here, even cells with almost undetectable mCherry exhibited the pan-nuclear signal as early as 8 h post-transfection, suggesting that very low CdtB expression is sufficient to induce significant DNA damage, whereas chromatin compaction is delayed by several hours, as classicaly observed during apoptotis in response to DNA damage. In accordance with this observation, AactCdtB direct expression has been shown to induce apoptosis after 48 h of transfection when the CdtB protein level is no longer detectable [46]. Finally, the CdtB protein transfection induces the DNA damage-related pattern of γH2AX signal through foci formation, that colocalise with 53BP1 (Fig 4D), a specific marker of the DNA damage response that is not involved during the apoptotic cascade [28]. Thus, the γH2AX signal observed reflects a direct response to the CdtB nuclease activity rather than an indirect effect associated to the phosphatase-related apoptotic induction.

Although the transfection assay allows to easily and rapidly discriminate active and catalytically inactive CdtB constructs, it does not permit quantifying CdtB nuclease activity due to the uncontrolled expression level. On the other hand, the direct transfection of CdtB recombinant proteins induced a time- and dose- dependent increase of γH2AX that require an intact CdtB catalytic site, supporting that this assay is suitable to quantify CdtB nuclease activity. Accordingly, CdtB direct transfection was shown to induce cell cycle arrest and apoptosis in a dose-dependent [33] and time-dependent [46] manner. Interestingly, γH2AX can be detected as soon as 1 hour post-transfection, which is consistent with microinjection experiments that have shown a CdtB nuclear localisation after 30 minutes [47], whilst it takes several hours to deliver CdtB to the nucleus *via* retrograde transport following holotoxin treatment. Therefore, CdtB transfection represents an easy and quantitative method to analyse CdtB nuclease activity without the prerequisite of host cell recognition imposed by the regulatory CdtA and CdtC subunits, implying that this strategy could be applied to directly compare the nuclease activity of CdtB subunits from various origins.

Using the same purified recombinants CdtBs, we have performed two unrelated experimental approaches dedicated to CdtB nuclease activity analysis. However, the activity measured with the plasmid digestion or the cell transfection assays should not be compared. Whilst the cellular system definitely distinguishes between catalytic mutants and WT CdtB subunits, indicating that the γH2AX signal quantification actually reflects CdtB specific activity, the biochemical assay did not, and rather seems to detect an artefactual activity. The lack of CdtB activity observable with the plasmid digestion assay could be explained by the need of a cellular cofactor for CdtB to be active, either a protein partner or a stronger affinity for chromatin instead of bare DNA. On the other hand, it is not excluded that contrary to DNase I, supercoiled double-stranded native DNA may not be a specific substrate for CdtB. Indeed, DNase I and CdtB belong to the exonuclease-endonuclease-phosphatase (EEP) family that includes other structure specific nucleases. For instance, APEX1 has been shown to be active on depurinated but not on native plasmid [48]. More globally, the endonuclease activity of many EEP family members depends on specific DNA alterations or structures [49–52]. In an effort to identify more specific substrates, we also tested the nuclease activity of CdtB on linear dsDNA and ssDNA, but were still unable to observe any activity that strictly depends on CdtB catalytic residues. Further studies will be needed to evaluate more precisely the CdtB nuclease activity on different DNA substrates.

In conclusion, this study shows that the biochemical approach classically performed to measure CdtB nuclease activity may not be sensitive enough to discriminate active from inactive proteins, and supports that cellular assay through transfection of recombinant CdtB subunits is a suitable alternative to directly compare their capacity to induce DNA double-strand breaks.

## Supporting information

S1 Fig Multiple sequence alignment of CdtB subunits from *H. ducreyi* and *E. coli* with bovine DNase I. Sequence similarity is highlighted in red, whereas sequence identity is shown as white letters on a red background. Residues mutated in this study are shown as white letters on a purple background (H153, conserved with the DNase I and involved in the catalytic site; D273, conserved with the DNase I and involved in the Mg2+ binding site). Conserved or highly similar residues are squared in blue. Top and bottom lines: secondary structure elements (arrows for β-strands and coils α-helices) of the CdtB subunit from *H*. *ducreyi* and bovine DNase I, respectively. Asterisks at the top of the alignment indicate every ten amino acids residues of HducCdtB.
(TIF)

S2 Fig DNA damage and apoptosis-related chromatin compaction in HeLa cells after 24 h of mCherry-CdtB expression. A. Representative images of mCherry-HducCdtB localisation, γH2AX immunofluorescence and DAPI staining from HeLa cells expressing WT or mutant (Hduc D273R or Ecol H153A) CdtB in fusion with mCherry. Immunostaining was performed 24 h after transfection. Cells with high expression (stars) of mutant mCherry-CdtB or with compacted chromatin (arrows) are indicated. Scale bar: 20 μm. B. Quantification of γH2AX positive HeLa cells left untransfected (NT) or expressing mutant CdtB (HducCdtB D273R or EcolCdtB H153A). Immunostaining was performed 24 h after transfection. Results present the mean ± SD of at least three independent experiments; statistical differences were analysed between transfected and non-transfected conditions (not significant).
(TIF)

S3 Fig DNA damage induction in U2OS cells after CDT holotoxin treatment, mCherry-CdtB expression or purified CdtB transfection. A. Representative images of γH2AX immunofluorescence and DAPI staining from U2OS cells treated with 20 ng/mL of WT or D273R HducCDT holotoxin or with 2.5 ng/mL of WT or H153A EcolCDT holotoxin for 24 h. Scale bar: 20 μm. B. Quantification of γH2AX positive U2OS cells left untreated (NT), treated with 20 ng/mL of WT or D273R HducCDT or with 2.5 ng/mL of WT or H153A EcolCDT for 24 h, represented as the mean fluorescence intensity per cell (normalised to 1 for the untreated condition) or as the proportion of γH2AX positive cells. Results present the mean ± SD of at least three independent experiments; statistical differences were analysed between treated and untreated conditions (** P < 0.01; **** P < 0.0001). C. Representative images of mCherry-HducCdtB localisation, γH2AX immunofluorescence and DAPI staining from U2OS cells expressing WT or mutant CdtB (HducCdtB D273R or EcolCdtB H153A) in fusion with mCherry. Immunostaining was performed 11 h after transfection. Cells with high expression (stars) or low expression (arrows) of WT mCherry-HducCdtB are indicated. Scale bar: 20 μm. D. Quantification of γH2AX positive U2OS cells left untransfected (NT), expressing WT or mutant CdtB (HducCdtB D273R or EcolCdtB H153A). Immunostaining was performed 11 h after transfection. Results present the mean ± SD of at least three independent experiments; statistical differences were analysed between transfected and non-transfected conditions (**** P < 0.0001). E. Representative images of γH2AX immunofluorescence and DAPI staining from U2OS cells transfected with 120 nM of WT or mutant (Hduc D273R or Ecol H153A) CdtB for 14 h. Scale bar: 20 μm. F. Quantification of γH2AX positive U2OS cells left untransfected (NT), transfected with 120 nM of WT or mutant (Hduc D273R or Ecol H153A) CdtB for 14 h, represented as the mean fluorescence intensity per cell (normalised to 1 for the untreated condition) or as the proportion of γH2AX positive cells. Results present the mean ± SD of at least three independent experiments; statistical differences were analysed between treated and untreated conditions (* P < 0.05; **** P < 0.0001) or between HducCdtB and EcolCdtB (## P < 0.01; #### P < 0.0001).
(TIF)

S4 Fig Dose-response analysis of the plasmid digestion assay with EcolCdtB and HducCdtB purified under native conditions. A. Dose-response analysis of the plasmid digestion assay in presence of WT or D273R HducCdtB. Agarose gel electrophoresis and quantification of supercoiled plasmid (250 ng) incubated with the indicated concentrations of WT or D273R HducCdtB for 10 min. M: molecular weight marker. B. CdtB concentration effect on the plasmid digestion assay in presence of WT or H153A EcolCdtB. Agarose gel electrophoresis and quantification of supercoiled plasmid (250 ng) incubated with the indicated concentrations of WT or D273R HducCdtB for 10 min. M: molecular weight marker. Arrows indicate plasmid conformation, either relaxed (R), linear (L) or supercoiled (S). For quantifications, the amount of each plasmid conformation is expressed as a proportion of the total plasmid content. Results present the mean ± SD of at least three independent experiments; statistical differences were analysed between every conditions and only mutant vs WT comparisons are shown (ns: not significant).
(TIF)

S5 Fig Size exclusion chromatography (SEC) of D273R HducCdtB. The curve represents the absorbance at 280 nm of the fractions eluted from the SEC column.
(TIF)

S6 Fig Mass spectrometry analysis. SEC fractions from the WT or D273R HducCdtB purifications were analysed after Mass spectrometry and peptides identified with Mascot algorithm. Data were searched against the complete *E*. *coli* proteome (SwissProt database). Fractions before the main elution peak (C2 for WT or B15 for D273R) did not contain any protein. The CdtB subunit was identified in the main SEC purification peaks (D4 for WT or D2 for D273R). The percentages of coverage (SC%) are respectively of 76.8 and 77.5% for WT and mutant CdtB. See SEC curves in Fig 3C for WT HducCdtB and S5 Fig for D273R HducCdtB.
(TIF)

S7 Fig Wild-type or catalytic mutant of EcolCdtB and HducCdtB present a similar double-stranded and single-stranded DNA digestion activity. A. Double-stranded DNA (dsDNA) and single-stranded DNA (ssDNA) substrates. Indicated amounts of undigested ssDNA and dsDNA were migrated on polyacrylamide gel electrophoresis. B. Ion content effect on dsDNA digestion assay by CdtB purified under native conditions. 100 nM of dsDNA were incubated with 50 ng of WT or mutant (Hduc D273R or Ecol H153A) CdtB purified under native conditions for 2 h in presence or in absence of Mg2+/Ca2+ buffer or with 1 μg of DNAse I for 10 min in presence of Mg2+/Ca2+ buffer. C. Ion content effect on ssDNA digestion assay by CdtB purified under native conditions. 100 nM of ssDNA were incubated with 50 ng of WT or mutant (Hduc D273R or Ecol H153A) CdtB purified under native conditions for 2 h in presence or in absence of Mg2+/Ca2+ buffer or with 1 μg of DNAse I for 10 min in presence of Mg2+/Ca2+ buffer. D. Ion content effect on ssDNA and dsDNA digestion assay by HducCdtB purified under denaturing conditions. 100 nM of ssDNA or dsDNA were incubated with 50 ng of WT or D273T HducCdtB purified under denaturing conditions for 2 h in presence or in absence of Mg2+/Ca2+ buffer.
(TIF)

S8 Fig DNA damage induction in HeLa cells after 24 or 48 h of mutant CdtB transfection. A. Representative images of γH2AX immunofluorescence and DAPI staining from HeLa cells transfected with 120 nM of mutant (Hduc D273R or Ecol H153A) CdtB for the indicated amount of time. Scale bar: 20 μm. B. Quantification of γH2AX positive HeLa cells left untransfected (NT), transfected with 120 nM of mutant (Hduc D273R or Ecol H153A) CdtB for the indicated amount of time, represented as the mean fluorescence intensity per cell (normalised to 1 for the untreated condition) or as the proportion of γH2AX positive cells. Results present the mean ± SD of at least three independent experiments; statistical differences were analysed between treated and untreated conditions (not significant) or between HducCdtB and EcolCdtB (not significant).
(TIF)

S9 Fig Raw data presenting whole gels and Western blots. A. Whole Western Blots of Fig 3C. Western blot analysis of the different SEC fractions shows CdtB in D4 and D2 fractions. B. Whole agarose gel electrophoresis used for nuclease assay quantification. Experimental conditions are identical to those described in the corresponding figures. NI: non incubated DNA; NT: DNA incubated without toxin; DR: D273R; HA: H153A. C. Whole polyacrylamide gel electrophoresis used in S7 Fig.
(TIF)

S1 File Minimal data set used for the publication. (XLSX)

## Abbreviations

CDT: Cytolethal Distending Toxin
DNase I: Deoxyribonuclease I
EcolCdtB: *Escherichia coli* CdtB
H2AX: Histone 2A variant X
HducCdtB: *Haemophilus ducreyi* CdtB
PBS: Phosphate-buffered saline
PAGE: Polyacrylamide Gel Electrophoresis
SEC: Size Exclusion Chromatography
SD: Standard deviation
WT: Wild Type
