# Supplementary figures and images for: Cell transfection of purified cytolethal distending toxin B subunits allows comparing their nuclease activity while plasmid degradation assay does not
Source: PLoS One. 2019 Mar 28;14(3):e0214313. doi: 10.1371/journal.pone.0214313 (PMC6438463; doi:10.1371/journal.pone.0214313)

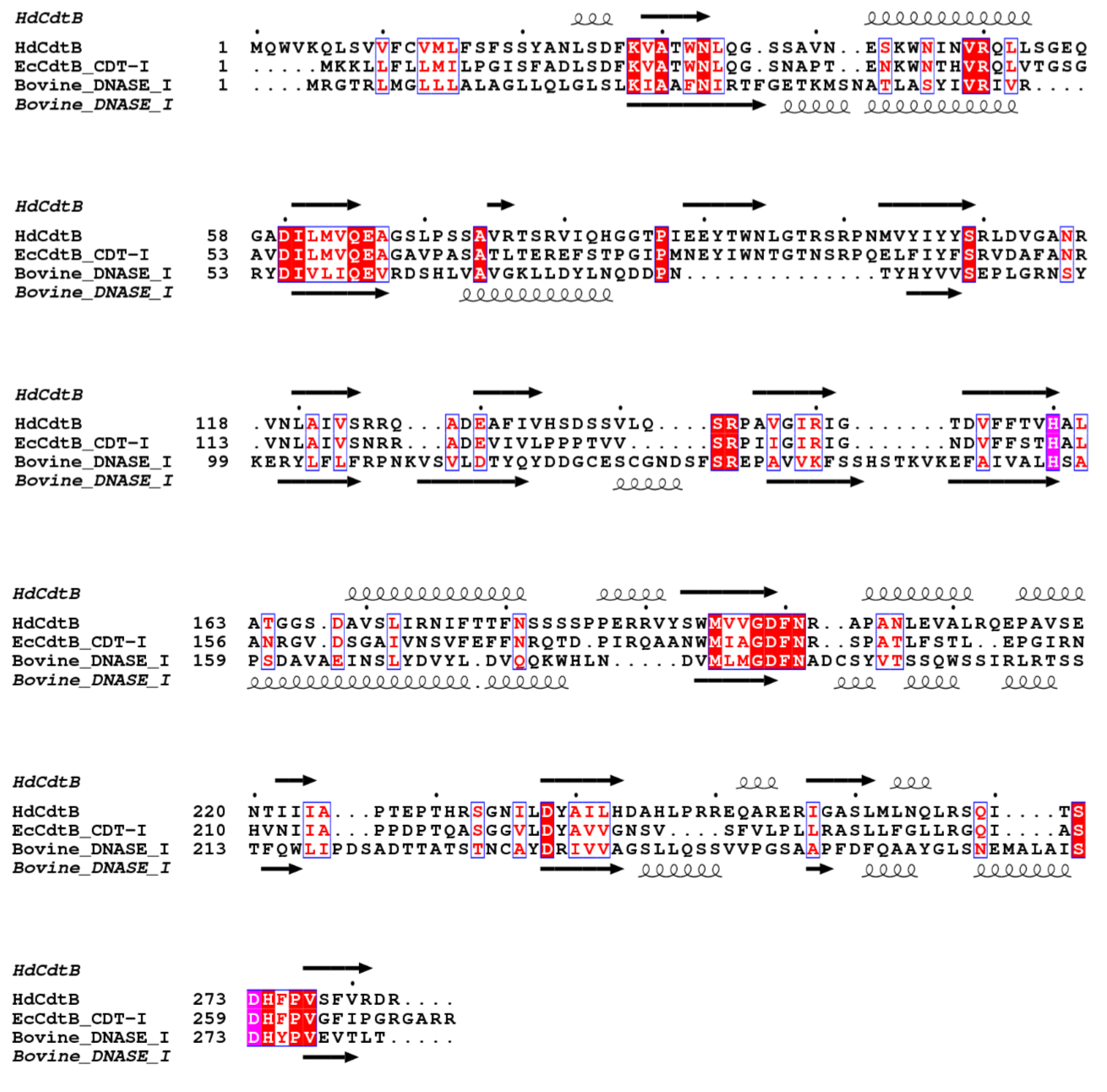

Supplement: S1 Fig — Sequence similarity is highlighted in red, whereas sequence identity is shown as white letters on a red background. Residues mutated in this study are shown as white letters on a purple background (H153, conserved with the DNase I and involved in the catalytic site; D273, conserved with the DNase I and involved in the Mg2+ binding site). Conserved or highly similar residues are squared in blue. Top and bottom lines: secondary structure elements (arrows for β-strands and coils α-helices) of the CdtB subunit from H. ducreyi and bovine DNase I, respectively. Asterisks at the top of the alignment indicate every ten amino acids residues of HducCdtB. (TIF) [file pone.0214313.s001.tif]

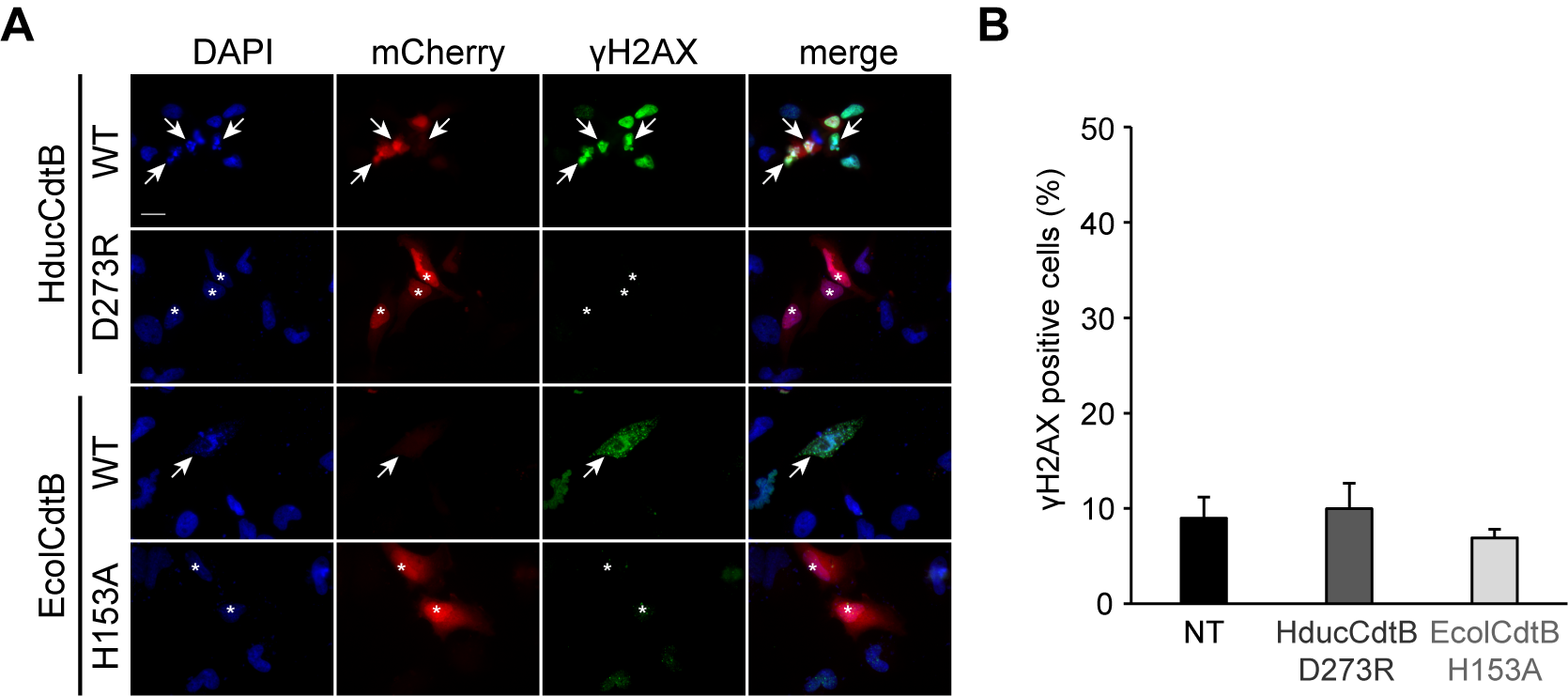

Supplement: S2 Fig — A. Representative images of mCherry-HducCdtB localisation, γH2AX immunofluorescence and DAPI staining from HeLa cells expressing WT or mutant (Hduc D273R or Ecol H153A) CdtB in fusion with mCherry. Immunostaining was performed 24 h after transfection. Cells with high expression (stars) of mutant mCherry-CdtB or with compacted chromatin (arrows) are indicated. Scale bar: 20 μm. B. Quantification of γH2AX positive HeLa cells left untransfected (NT) or expressing mutant CdtB (HducCdtB D273R or EcolCdtB H153A). Immunostaining was performed 24 h after transfection. Results present the mean ± SD of at least three independent experiments; statistical differences were analysed between transfected and non-transfected conditions (not significant). (TIF) [file pone.0214313.s002.tif]

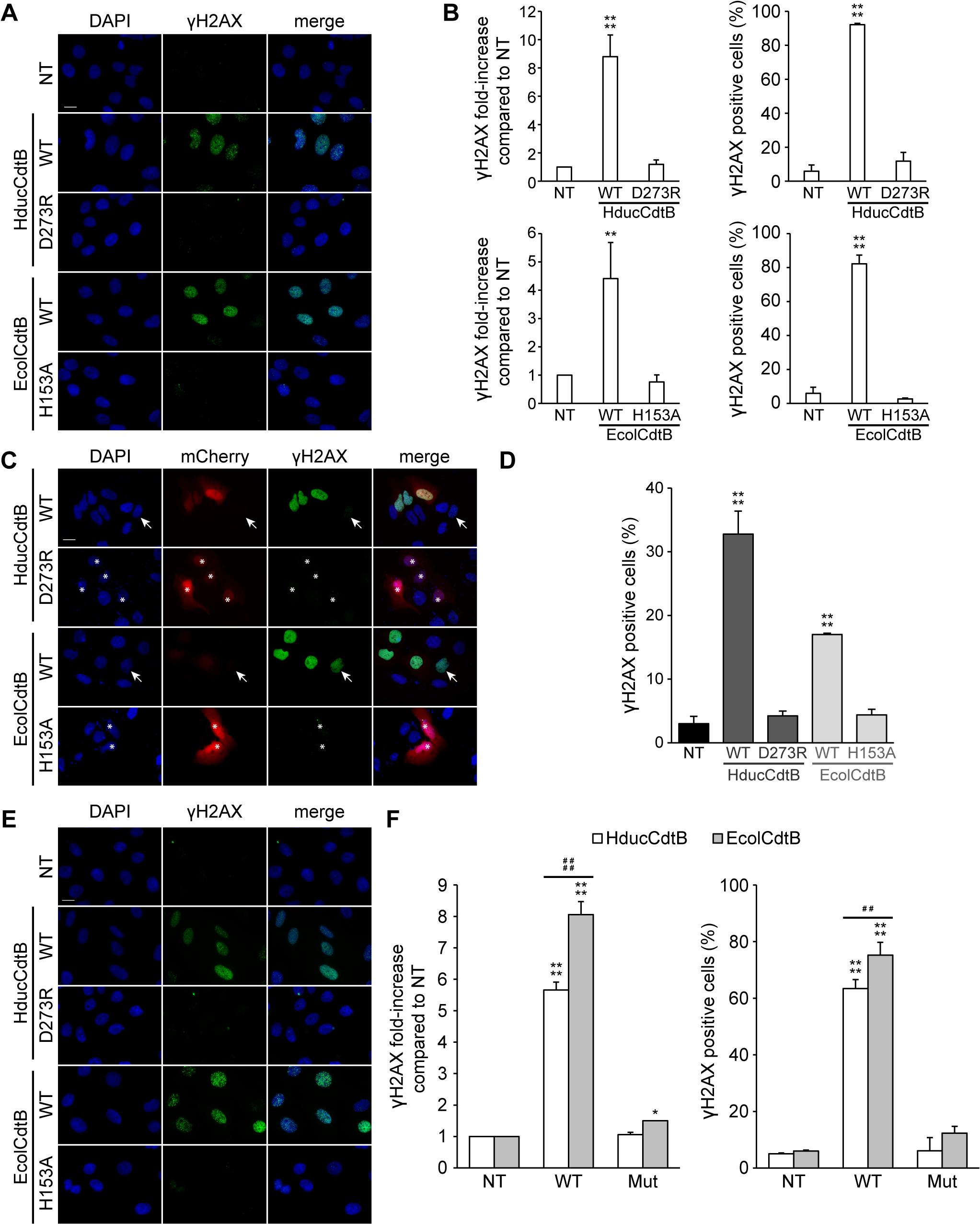

Supplement: S3 Fig — A. Representative images of γH2AX immunofluorescence and DAPI staining from U2OS cells treated with 20 ng/mL of WT or D273R HducCDT holotoxin or with 2.5 ng/mL of WT or H153A EcolCDT holotoxin for 24 h. Scale bar: 20 μm. B. Quantification of γH2AX positive U2OS cells left untreated (NT), treated with 20 ng/mL of WT or D273R HducCDT or with 2.5 ng/mL of WT or H153A EcolCDT for 24 h, represented as the mean fluorescence intensity per cell (normalised to 1 for the untreated condition) or as the proportion of γH2AX positive cells. Results present the mean ± SD of at least three independent experiments; statistical differences were analysed between treated and untreated conditions (** P < 0.01; **** P < 0.0001). C. Representative images of mCherry-HducCdtB localisation, γH2AX immunofluorescence and DAPI staining from U2OS cells expressing WT or mutant CdtB (HducCdtB D273R or EcolCdtB H153A) in fusion with mCherry. Immunostaining was performed 11 h after transfection. Cells with high expression (stars) or low expression (arrows) of WT mCherry-HducCdtB are indicated. Scale bar: 20 μm. D. Quantification of γH2AX positive U2OS cells left untransfected (NT), expressing WT or mutant CdtB (HducCdtB D273R or EcolCdtB H153A). Immunostaining was performed 11 h after transfection. Results present the mean ± SD of at least three independent experiments; statistical differences were analysed between transfected and non-transfected conditions (**** P < 0.0001). E. Representative images of γH2AX immunofluorescence and DAPI staining from U2OS cells transfected with 120 nM of WT or mutant (Hduc D273R or Ecol H153A) CdtB for 14 h. Scale bar: 20 μm. F. Quantification of γH2AX positive U2OS cells left untransfected (NT), transfected with 120 nM of WT or mutant (Hduc D273R or Ecol H153A) CdtB for 14 h, represented as the mean fluorescence intensity per cell (normalised to 1 for the untreated condition) or as the proportion of γH2AX positive cells. Results present the mean ± SD of at least th [file pone.0214313.s003.tif]

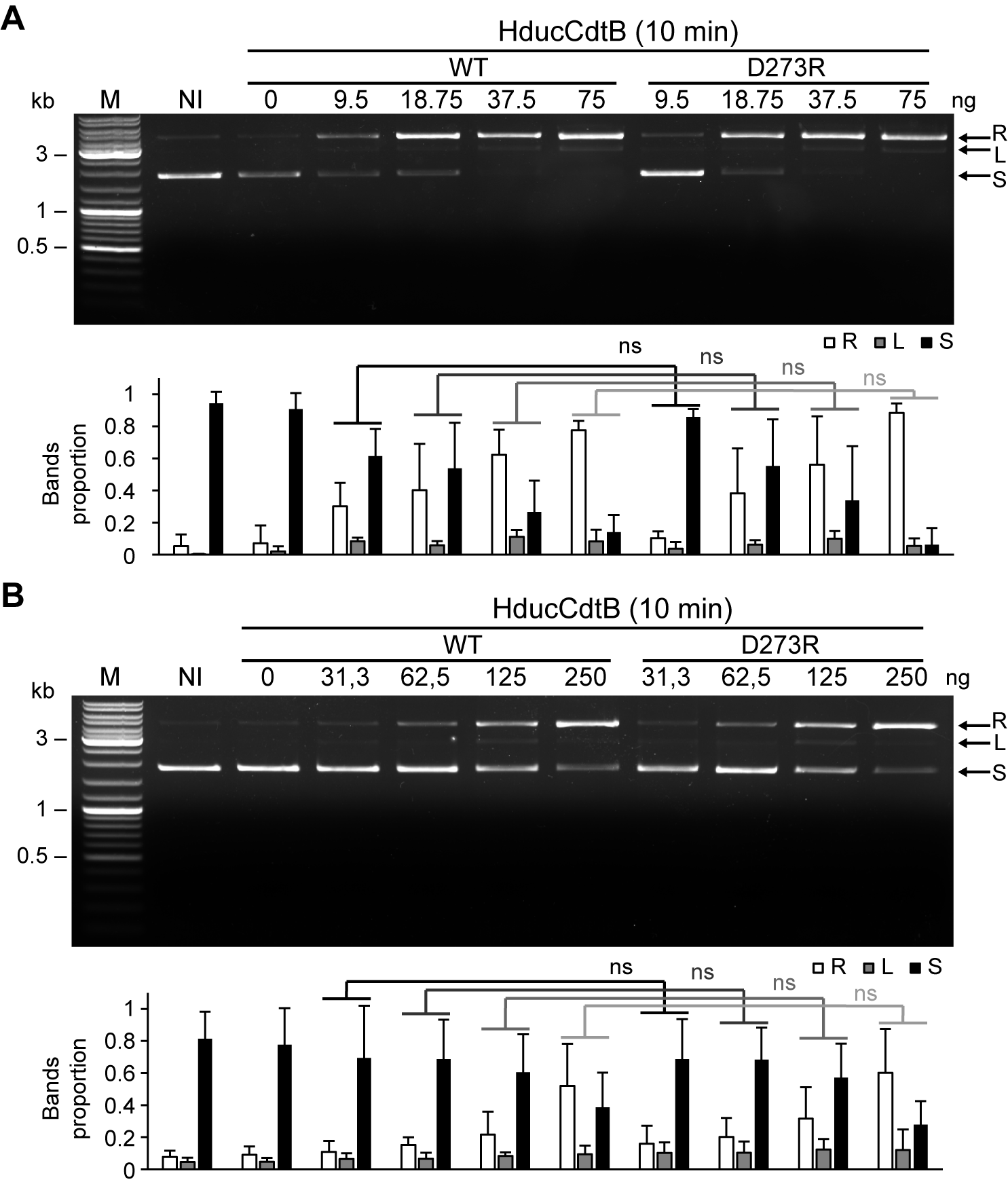

Supplement: S4 Fig — A. Dose-response analysis of the plasmid digestion assay in presence of WT or D273R HducCdtB. Agarose gel electrophoresis and quantification of supercoiled plasmid (250 ng) incubated with the indicated concentrations of WT or D273R HducCdtB for 10 min. M: molecular weight marker. B. CdtB concentration effect on the plasmid digestion assay in presence of WT or H153A EcolCdtB. Agarose gel electrophoresis and quantification of supercoiled plasmid (250 ng) incubated with the indicated concentrations of WT or D273R HducCdtB for 10 min. M: molecular weight marker. Arrows indicate plasmid conformation, either relaxed (R), linear (L) or supercoiled (S). For quantifications, the amount of each plasmid conformation is expressed as a proportion of the total plasmid content. Results present the mean ± SD of at least three independent experiments; statistical differences were analysed between every conditions and only mutant vs WT comparisons are shown (ns: not significant). (TIF) [file pone.0214313.s004.tif]

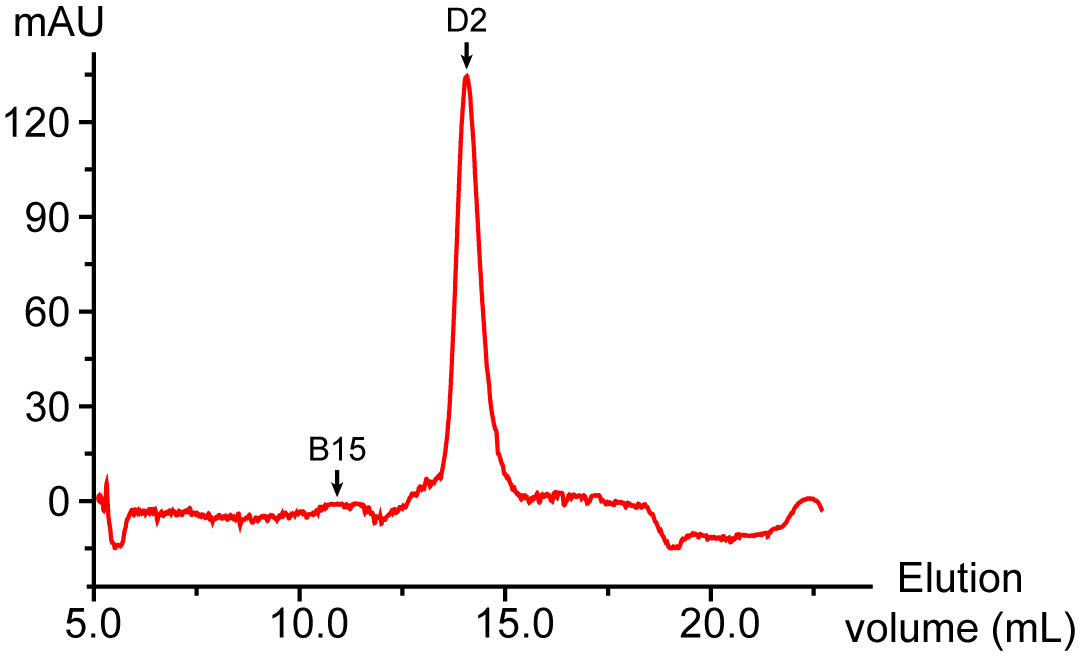

Supplement: S5 Fig — The curve represents the absorbance at 280 nm of the fractions eluted from the SEC column. (TIF) [file pone.0214313.s005.tif]

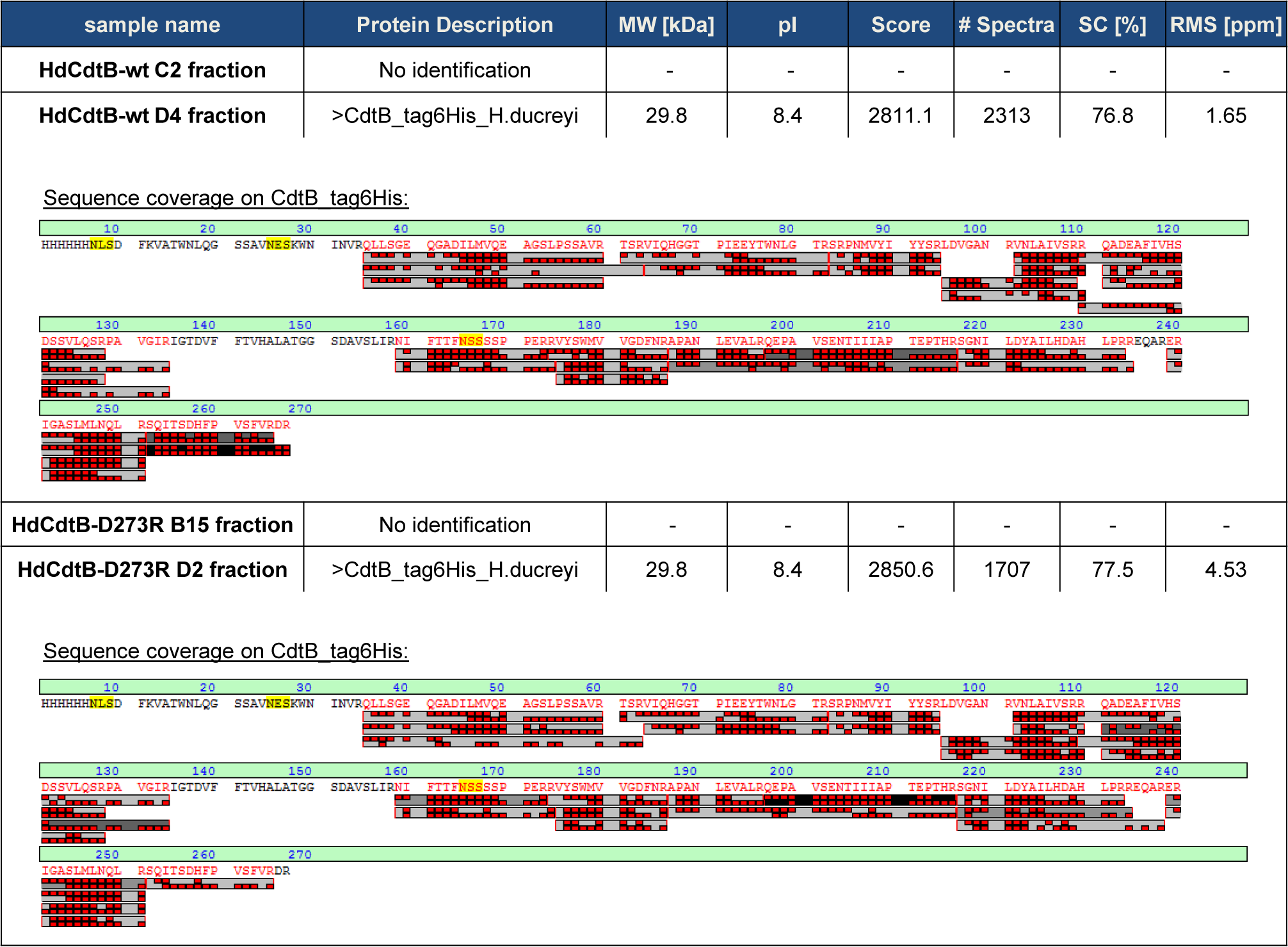

Supplement: S6 Fig — SEC fractions from the WT or D273R HducCdtB purifications were analysed after Mass spectrometry and peptides identified with Mascot algorithm. Data were searched against the complete E. coli proteome (SwissProt database). Fractions before the main elution peak (C2 for WT or B15 for D273R) did not contain any protein. The CdtB subunit was identified in the main SEC purification peaks (D4 for WT or D2 for D273R). The percentages of coverage (SC%) are respectively of 76.8 and 77.5% for WT and mutant CdtB. See SEC curves in Fig 3C for WT HducCdtB and S5 Fig for D273R HducCdtB. (TIF) [file pone.0214313.s006.tif]

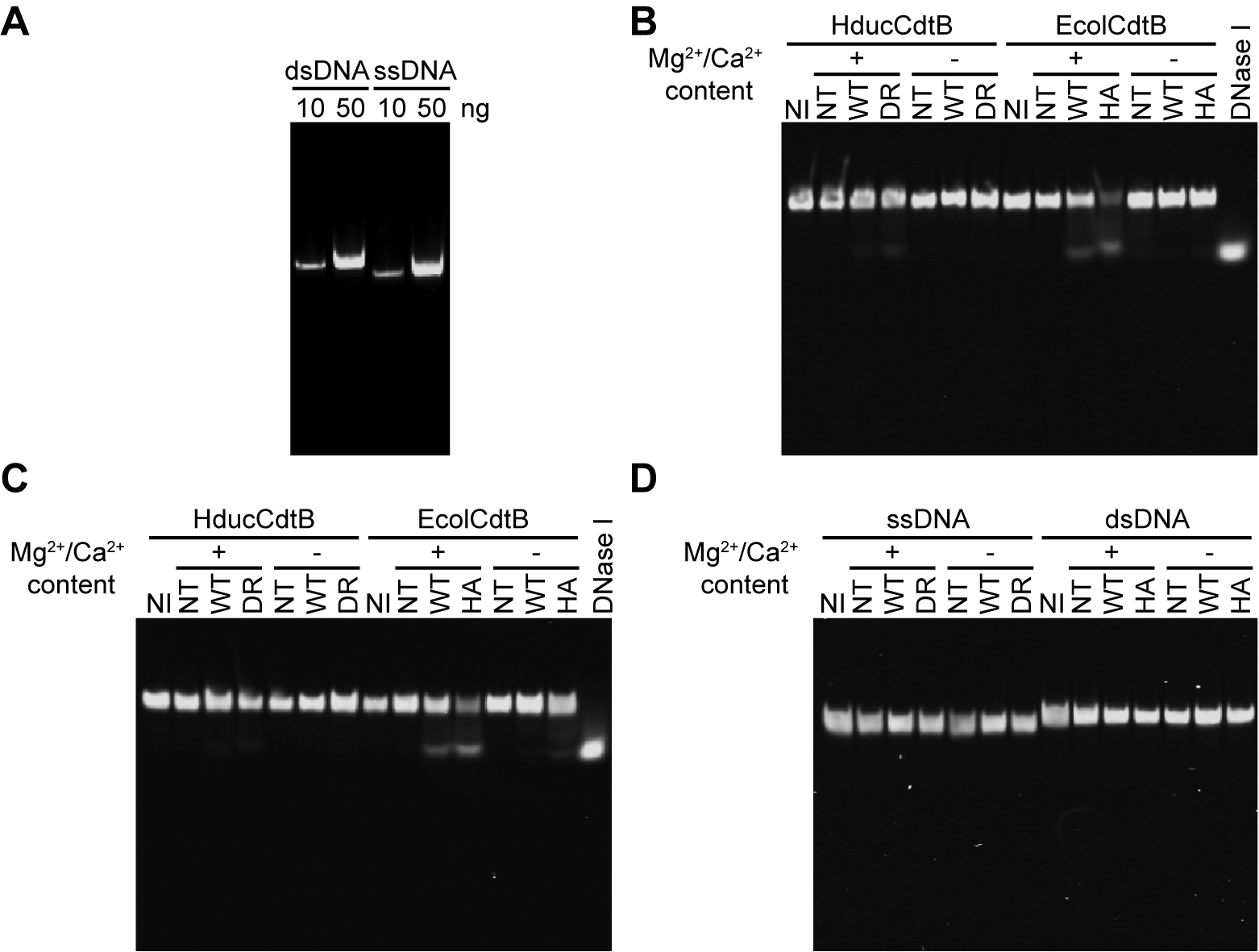

Supplement: S7 Fig — A. Double-stranded DNA (dsDNA) and single-stranded DNA (ssDNA) substrates. Indicated amounts of undigested ssDNA and dsDNA were migrated on polyacrylamide gel electrophoresis. B. Ion content effect on dsDNA digestion assay by CdtB purified under native conditions. 100 nM of dsDNA were incubated with 50 ng of WT or mutant (Hduc D273R or Ecol H153A) CdtB purified under native conditions for 2 h in presence or in absence of Mg2+/Ca2+ buffer or with 1 μg of DNAse I for 10 min in presence of Mg2+/Ca2+ buffer. C. Ion content effect on ssDNA digestion assay by CdtB purified under native conditions. 100 nM of ssDNA were incubated with 50 ng of WT or mutant (Hduc D273R or Ecol H153A) CdtB purified under native conditions for 2 h in presence or in absence of Mg2+/Ca2+ buffer or with 1 μg of DNAse I for 10 min in presence of Mg2+/Ca2+ buffer. D. Ion content effect on ssDNA and dsDNA digestion assay by HducCdtB purified under denaturing conditions. 100 nM of ssDNA or dsDNA were incubated with 50 ng of WT or D273T HducCdtB purified under denaturing conditions for 2 h in presence or in absence of Mg2+/Ca2+ buffer. (TIF) [file pone.0214313.s007.tif]

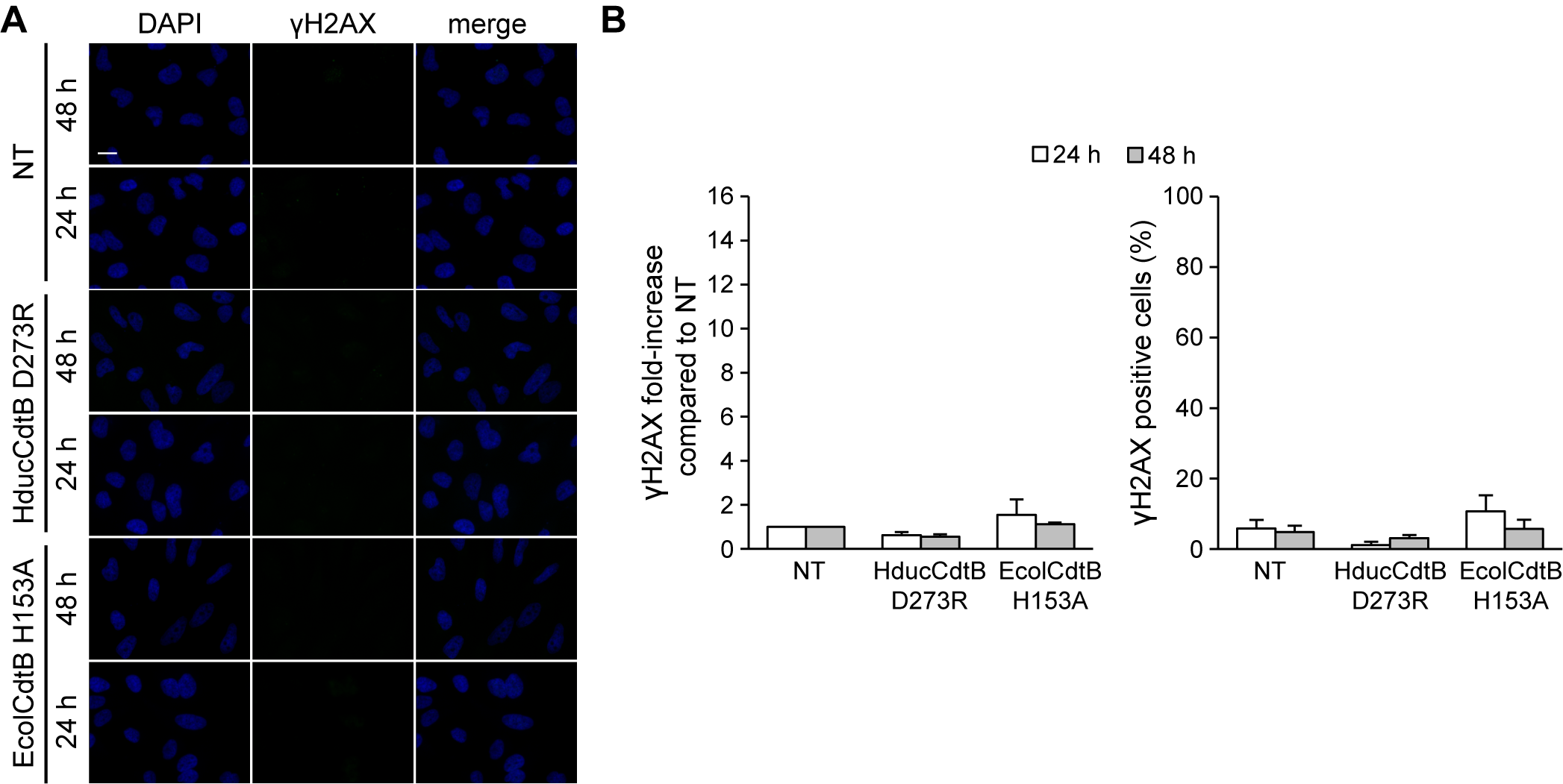

Supplement: S8 Fig — A. Representative images of γH2AX immunofluorescence and DAPI staining from HeLa cells transfected with 120 nM of mutant (Hduc D273R or Ecol H153A) CdtB for the indicated amount of time. Scale bar: 20 μm. B. Quantification of γH2AX positive HeLa cells left untransfected (NT), transfected with 120 nM of mutant (Hduc D273R or Ecol H153A) CdtB for the indicated amount of time, represented as the mean fluorescence intensity per cell (normalised to 1 for the untreated condition) or as the proportion of γH2AX positive cells. Results present the mean ± SD of at least three independent experiments; statistical differences were analysed between treated and untreated conditions (not significant) or between HducCdtB and EcolCdtB (not significant). (TIF) [file pone.0214313.s008.tif]

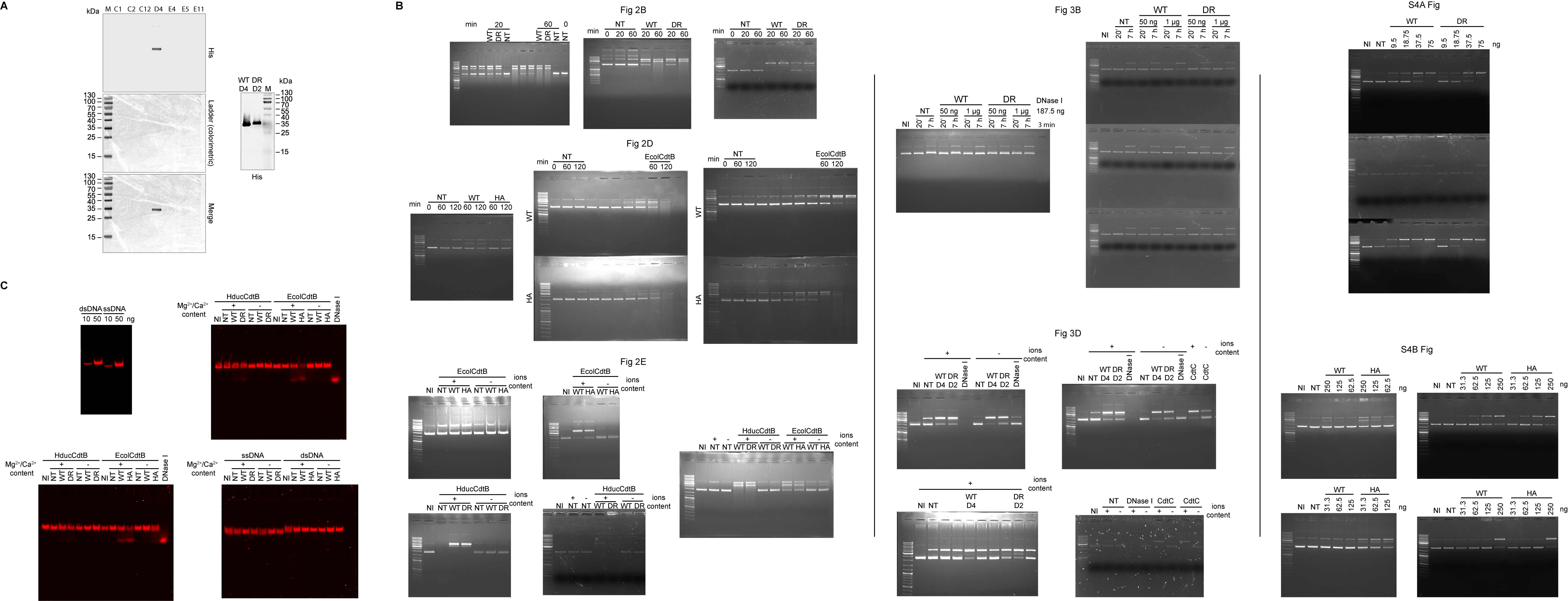

Supplement: S9 Fig — A. Whole Western Blots of Fig 3C. Western blot analysis of the different SEC fractions shows CdtB in D4 and D2 fractions. B. Whole agarose gel electrophoresis used for nuclease assay quantification. Experimental conditions are identical to those described in the corresponding figures. NI: non incubated DNA; NT: DNA incubated without toxin; DR: D273R; HA: H153A. C. Whole polyacrylamide gel electrophoresis used in S7 Fig. (TIF) [file pone.0214313.s009.tif]
